# *RNF43* G659fs is an oncogenic colorectal cancer mutation and sensitizes tumor cells to PI3K/mTOR inhibition

**DOI:** 10.1038/s41467-022-30794-7

**Published:** 2022-06-08

**Authors:** Lishan Fang, Dane Ford-Roshon, Max Russo, Casey O’Brien, Xiaozhe Xiong, Carino Gurjao, Maximilien Grandclaudon, Srivatsan Raghavan, Steven M. Corsello, Steven A. Carr, Namrata D. Udeshi, James Berstler, Ewa Sicinska, Kimmie Ng, Marios Giannakis

**Affiliations:** 1 Dana-Farber Cancer Institute, Harvard Medical School, Boston, MA USA; 2 Broad Institute of MIT and Harvard, Cambridge, MA USA; 3 Medical Research Center, The Eighth Affiliated Hospital, Sun Yat-sen University, Shenzhen, China; 4 Department of Urology, Boston Children’s Hospital, Boston, MA USA; 5 Department of Microbiology, Blavatnik Institute, Harvard Medical School, Boston, MA USA

**Keywords:** Colorectal cancer, Molecular biology

## Abstract

The *RNF43*_p.G659fs mutation occurs frequently in colorectal cancer, but its function remains poorly understood and there are no specific therapies directed against this alteration. In this study, we find that *RNF43*_p.G659fs promotes cell growth independent of Wnt signaling. We perform a drug repurposing library screen and discover that cells with *RNF43*_p.G659 mutations are selectively killed by inhibition of PI3K signaling. PI3K/mTOR inhibitors yield promising antitumor activity in *RNF43*^659mut^ isogenic cell lines and xenograft models, as well as in patient-derived organoids harboring *RNF43*_p.G659fs mutations. We find that RNF43^659mut^ binds p85 leading to increased PI3K signaling through p85 ubiquitination and degradation. Additionally, RNA-sequencing of *RNF43*^659mut^ isogenic cells reveals decreased interferon response gene expression, that is reversed by PI3K/mTOR inhibition, suggesting that *RNF43*^659mut^ may alter tumor immunity. Our findings suggest a therapeutic application for PI3K/mTOR inhibitors in treating *RNF43*_p.G659fs mutant cancers.

## Abstract (web summary)

The RNF43 G659fs mutation occurs frequently in colorectal cancer, but its function remains poorly understood. In this study, the authors show that RNF43 G659fs is an oncogenic colorectal cancer mutation and sensitizes tumor cells to PI3K/mTOR inhibition.

## Introduction

Colorectal cancer (CRC) is the second leading cause of cancer-related mortality and the third most commonly diagnosed cancer worldwide with a growing incidence rate among younger patients^1,2^. Despite advances in treatment strategies for CRC, the outcomes for patients with CRC have only modestly improved^3^. Thus, there is a pressing need to develop novel treatment options for patients with this disease. Despite extensive genomic profiling of CRC^4–6^, the role of CRC genetic variants in therapeutic decisions remains limited largely due to the fact that the carcinogenic role of relevant mutations is inadequately characterized^7–9^. Therefore, determining the specific function of key genetic variants is critical to facilitate new targeted therapeutic approaches, and ultimately improve patient outcomes.

*RNF43* (Ring Finger Protein 43) encodes a Ring finger E3 ubiquitin ligase which acts as a negative regulator of the Wnt signaling pathway through the degradation of the Wnt receptor, Frizzled^10^. Thus, *RNF43* loss-of-function renders tumor cells dependent on secreted Wnt ligands for survival^10–12^. Inactivating mutations in *RNF43* were reported to confer susceptibility to pharmacologic inhibitors that target the Wnt-specific acyltransferase porcupine (PORCN) in pancreatic ductal adenocarcinoma^13^, gastric cancer^14^ and CRC^15^. Intriguingly, two truncating mutations, *RNF43*_p.G659fs and *RNF43*_p.R117fs, have been observed with high frequency in CRC^6,16,17^. The more frequent G659fs mutation is found in ~8% of patients with CRC and is enriched in microsatellite-instability high cancers^16^. However, the functional consequences of *RNF43* mutations on Wnt signaling as well as colorectal carcinogenesis remain controversial, especially for p.G659fs. Previous studies have showed that the *RNF43*_p.G659fs mutation remains fully functional in promoting Frizzled degradation and attenuating Wnt signaling and is thus unlikely to confer Wnt-dependency onto CRC^18,19^. In line with this, Wetering et al. showed that a CRC organoid which harbors a *RNF43_*p.G659fs mutation was insensitive to two selective PORCN inhibitors, WNT974 and IWP2^15^. Indeed, PORCN inhibitors are currently in clinical trials, however, patients with *RNF43*_p.G659fs mutations are not eligible due to insufficient evidence supporting Wnt ligand-dependency for these tumors. Consequently, although *RNF43*_p.G659fs truncation is a frequent mutational event in CRC, there are currently no therapeutics targeting it. Thus, mechanistic insights and identification of novel therapeutics for the treatment of patients whose tumors harbor the *RNF43*_p.G659fs mutation are crucial.

In this study, we provide molecular evidence that the *RNF43*_p.G659fs mutation drives CRC in a Wnt-independent manner. We find that *RNF43*_p.G659fs mutant colorectal tumor cells activate PI3K/AKT signaling and that PI3K inhibitors selectively target *RNF43*_p.G659fs mutant tumor cells. Mechanistically, we discover that *RNF43*_p.G659fs interacts with p85 (Phosphoinositide-3-Kinase Regulatory Subunit 1) to increase PI3K (Phosphoinositide-3-Kinase)/AKT signaling. Additionally, we associate *RNF43_*p.G659fs mutations with decreased interferon response. Our results support PI3K inhibition as a therapeutic option for patients whose tumors harbor the *RNF43*_p.G659fs mutation.

## Results

### *RNF43*^659mut^ has a Wnt-independent carcinogenic role

**Fig. 1:**
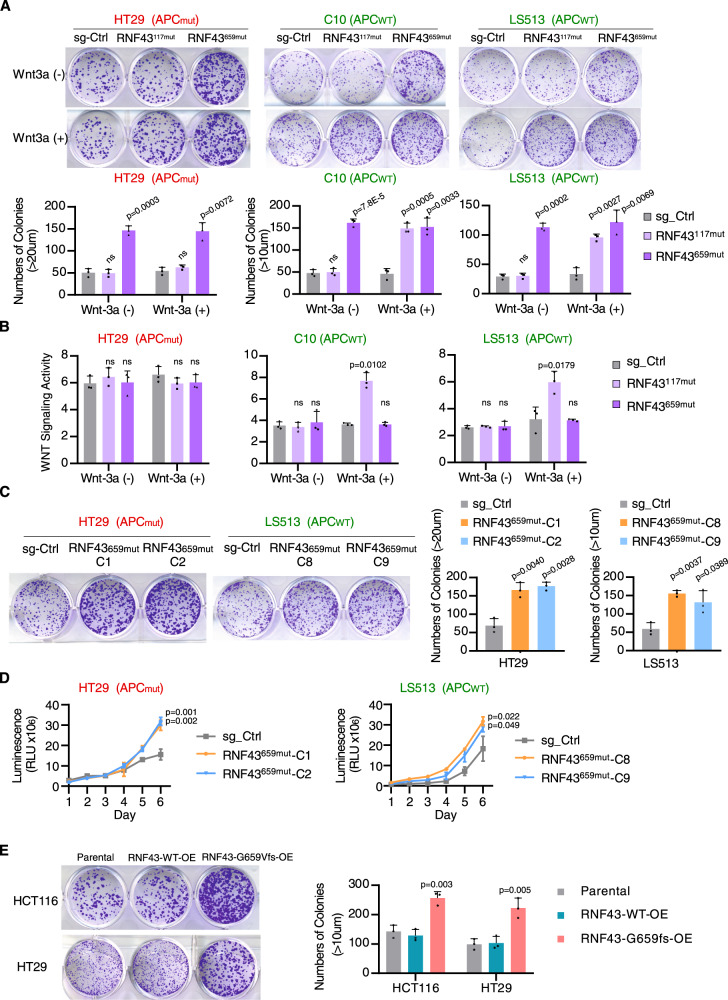
*RNF43*^659mut^ promotes cell growth and viability independent of Wnt signaling. **A** Representative image (upper panel) and quantification (lower panel) of colony formation assays in sgRNA-control, *RNF43*^117mut^ and *RNF43*^659mut^ CRISPR-edited HT29, C10 and LS513 CRC cells in the absence (−) or presence (+) of exogenous Wnt-3a ligand. **B** Luciferase reporter assay of Wnt signaling activity in indicated CRISPR-edited cells. **C** Representative images (left panel) and quantification (right panel) of colony formation assay in two single *RNF43*^659mut^ clones in each of HT29 and LS513 cell lines, compared to non-targeting sgRNA-control. **D** Cell viability assay for control and *RNF43*^659mut^ edited single clones of HT29 and LS513 cells. **E** Representative images and quantification of colony formation assays with *RNF43*-wildtype (WT) and *RNF43*-659fs*41 overexpression (OE) in HCT116 and HT29 cells. Data are the mean ± SD. Two-sided Student’s *t* test (*n* = 3 independent experiments in triplicate). Source data are provided as a Source Data file.

*RNF43* is recurrently mutated at two residues, p.R117fs and p.G659fs in CRC^16^. To evaluate whether these *RNF43* hot-spot frameshift mutations functionally contribute to tumor progression, we selected several CRC cell lines with wildtype *RNF43* and *CTNNB1* and either wildtype or mutant *APC*^20^, namely C10 (*APC*^WT^), LS513 (*APC*^WT^) and HT29 (*APC*^mut^) and utilized CRISPR-Cas9 to genetically edit the target sites of RNF43-117aa (*RNF43*^117mut^) and RNF43-659aa (*RNF43*^659mut^) (Supplementary Fig. 1a). Cell growth was initially assessed through colony formation assays with and without addition of Wnt-3a ligand. Compared to sgRNA controls, the addition of Wnt-3a demonstrated increased cell growth in *RNF43*^117mut^ cells in both *APC*^WT^ cell lines (C10 and LS513) (Fig. 1A). However, no significant difference was observed in the *APC*^WT^ cells without Wnt-3a or in the *APC*^*mut*^ cell line (HT29) with intrinsic Wnt activation (Fig. 1A) indicating the function of *RNF43*^117mut^ was Wnt context and ligand-dependent. Intriguingly, we found that *RNF43*^659mut^ significantly increased colony formation irrespective of the presence of Wnt-3a or *APC* mutation status across all three CRC lines (Fig. 1A). To further assess Wnt activation in *RNF43*^117mut^ and *RNF43*^659mut^ cell growth, we carried out a TOP/FOP luciferase reporter assay to measure Wnt signaling activity. As illustrated in Fig. 1B, *RNF43*^117mut^ significantly increased Wnt signaling upon Wnt-3a supplementation in the *APC*^WT^ C10 and LS513 cells. However, *RNF43*^659mut^ had no increased Wnt activity compared to control regardless of APC status or Wnt-3a supplementation suggesting intact suppression of Wnt signaling by *RNF43*^659mut^ (Fig. 1B). Consistent with this finding, we found no change in the expression and phosphorylation of LRP6, GSK3β, CK1, or CTNNB1 in isogenic HT29 *RNF43*^659mut^ cells (Supplementary Fig. 2). Colony formation assays of nine distinct *RNF43*^659mut^ single clones derived from HT29 and LS513 confirmed increased cell growth (Fig. 1C, D and Supplementary Fig. 1b, c). Indicated single clones were used in subsequent experiments.

To assess for a gain of function with *RNF43*_p.G659Vfs, we overexpressed V5-tagged *RNF43*-wildtype and *RNF43*_p.G659Vfs protein into the HCT116 (*RNF43*^117mut^) and HT29 (*RNF43*^WT^) cell lines. Consistent with the observation in the CRISPR-edited isogenic cell lines, overexpression of *RNF43*_p.G659Vfs caused a significant growth increase compared to either *RNF43*-wildtype or the non-transfected control in both HCT116 and HT29 cell lines, confirming the oncogenic role for *RNF43*^659mut^ (Fig. 1E). Collectively, these results provide evidence that *RNF43*_p.G659Vfs has a functional role in carcinogenesis via a Wnt-independent manner.

### *RNF43*^659mut^ activates PI3K/AKT signaling and is vulnerable to PI3K/mTOR inhibitors

**Fig. 2:**
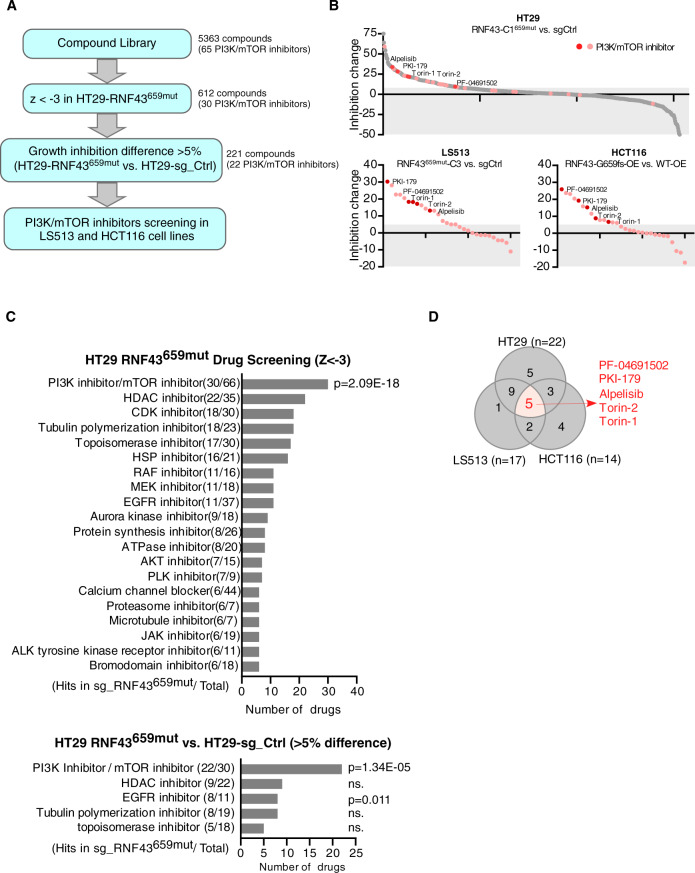
Repurposing library screening to identify compounds targeting *RNF43*_659fs. **A** Flowchart of Repurposing Library compound screening and selection criteria for candidate drug identification. **B** Quantification of selective inhibition of library compounds in HT29 (top), and validation of PI3K/mTOR inhibitor compounds in LS513 *RNF43*^659mut^ edited cells and HCT116 *RNF43*^*G*659fs^ overexpressing (OE) cells (bottom). Red data points indicate PI3K/mTOR inhibitors. **C** Pathway enrichment analysis of repurposing library compound screening in the initial screening population of HT29 *RNF43*^659mut^ with a *Z* value less than −3 (top, among 612 significant drugs) and compounds that had >5% killing between HT29 *RNF43*^659mut^ and HT29 ctrl (bottom, among 221 significant drugs). Two-sided Pearson’s *χ*^2^ test. **D** Venn diagram illustration of PI3K/mTOR significant candidate compound overlap (red) in HT29, LS513 *RNF43*^659mut^ edited cells and HCT116 *RNF43*^659mut^ overexpressing cells with *n* the number of compounds from the primary screen that validated in the secondary screen for each cell line. Source data are provided as a Source Data file.

In order to discover potential therapeutic vulnerabilities of *RNF43*^659mut^ CRC cells, we performed a comprehensive drug screen to identify small molecules that selectively inhibit *RNF43*^659mut^ cells in HT29 CRISPR-edited isogenic cell lines. We tested the Broad Institute’s Repurposing Library^21^, which contained a set of 5363 compounds at different phases of preclinical and clinical development. For the primary screen, we identified 612 compounds that reduced the viability of *RNF43*^659mut^ with a *z*-score < -3 compared to DMSO-treated controls (Fig. 2A and Supplementary Data 1). Out of these, 221 compounds had >5% killing in *RNF43*^659mut^ compared to *RNF43*^WT^ isogenic cells (Fig. 2A, B). Remarkably, among the 612 and 221 compound lists, we identified 30 and 22 drugs, respectively, targeting the PI3K/mTOR pathway (Fig. 2A and Supplementary Table 1). This pathway was significantly enriched among the repurposing library compounds that selectively target *RNF43*^659mut^ over DMSO-treated and isogenic controls (Fig. 2C). We selected PI3K/mTOR compounds (*n* = 30) for a secondary screen at nine doses (0 to 10 uM) in LS513 *RNF43*^659mut^ CRISPR-edited cells and HCT116 *RNF43*^*G*659fs^-overepressing (OE) cells (Fig. 2B). We observed that five compounds, alpelisib, PF-04691502, PKI-179, Torin-1, and Torin-2, led to significant selective killing in all three cell lines (Fig. 2B, D).

**Fig. 3:**
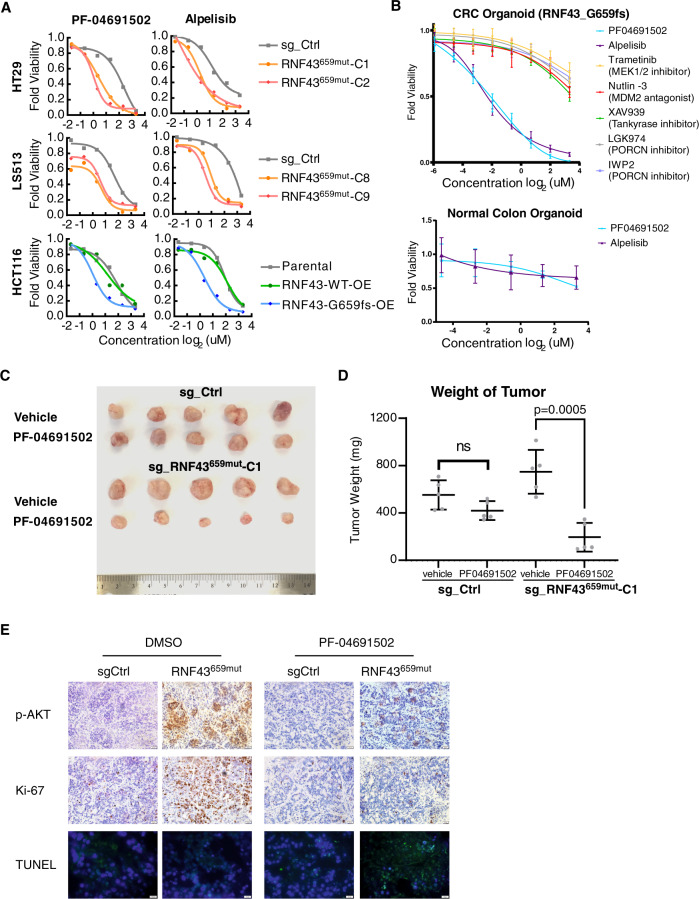
Assessment of PI3K/mTOR compound efficacy in CRC *RNF43*_659fs preclinical models. **A** Alpelisib and PF-04691502 dose-response curves in either *RNF43*^659mut^ edited cells (HT29, LS513) or ectopically expressing (OE) *RNF43*_G659Vfs*41 cells (HCT116) compared to controls. Experiments were performed in triplicate. **B** Dose-response curve of alpelisib and PF-04691502, along with an MDM2 antagonist and MEK, tankyrase, and PORCN inhibitors, in patient-derived CRC organoids harboring *RNF43*_G659fs and normal colon organoids. **C** Image representation of mouse tumor size after placebo or PF-04691502 treatment in cell-derived xenografts harboring either HT29 sg_Ctrl or HT29 sg_*RNF43*^659mut^. **D** Quantification of tumor weight after placebo or PF-04691502 treatment in cell-derived xenograft harboring either HT29 sg_Ctrl or HT29 sg_*RNF43*^659mut^. Data are the mean ± SD. Two-sided Student’s *t* test (*n* = 5 mice/group). **E** Representative immunohistochemistry images of p-AKT (S473), Ki-67 and TUNEL-positive cells in HT29_sg-Ctrl or HT29_RNF43^659mut^-C1 tumors from mice treated with vehicle or PF-04691502. Scale bar 10 μm. Source data are provided as a Source Data file.

We further characterized and optimized the dose-response curve with alpelisib, PF-04691502, PKI-179, Torin-1, and Torin-2 in *RNF43*^659mut^ HT29 and LS513 cells, as well as HCT116 *RNF43*^*G*659fs^-OE cells. All five compounds exhibited potent and selective toxicity toward *RNF43*^659mut^ cells and overexpressing *RNF43*^659mut^ cells, but not toward sgRNA-control cells or wildtype-overexpressing cells (Fig. 3A and Supplementary Fig. 3). Further testing of alpelisib, which is FDA-approved for breast cancer treatment, and PF-04691502, currently in clinical trials, showed selective killing of CRC organoids harboring the *RNF43*_p.G659Vfs mutation (Fig. 3B). Through analysis of TCGA CRC whole-transcriptome (RNA-Seq) data, we also observed that tumors with *RNF43*^659mut^ had an increased PI3Ki sensitivity signature^22^ (Supplementary Fig. 4). We further evaluated the effectiveness of PI3K/mTOR inhibition against the *RNF43_*p.G659fs mutation in HT29 cell line-derived xenograft (CDX) models using sg_Ctrl mice and *RNF43*^659mut^ mice. PF-04691502 treatment over 28 days was able to reduce tumor growth in *RNF43*^659mut^ CDX mice compared to the placebo group while there was no significant change in the sg_Ctrl mice (Fig. 3C, D). These results provide evidence that PI3K/mTOR inhibitors can target tumors harboring *RNF43*^659mut^ mutations.

### *RNF43*^659mut^ activates PI3K/AKT signaling through RNF43^659mut^-p85 interaction

**Fig. 4:**
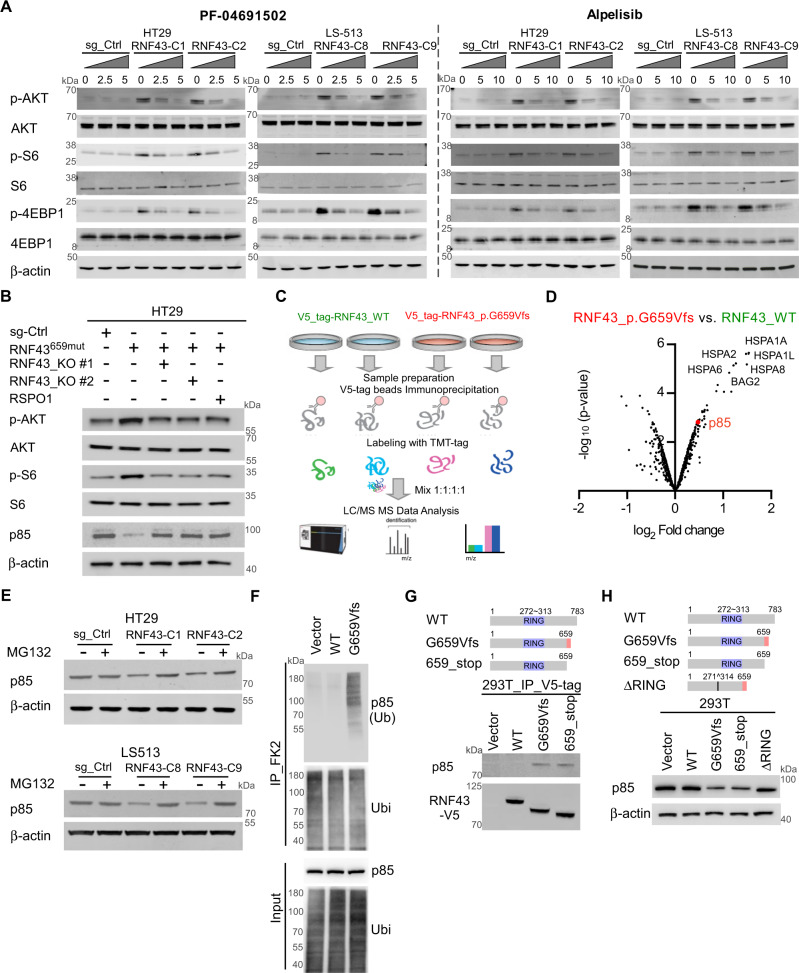
PI3K/AKT activation by *RNF43*_G659Vfs and quantitative proteomic profiling of *RNF43*_G659Vfs. **A** Expression of p-AKT, p-S6, and p-4EBP1 in *RNF43*^659mut^ edited and sg_Ctrl isogenic HT29 and LS513 cells after 48 h treatment with PF-04691502 (left) or alpelisib (right). β-Actin was used as a loading control. **B** p-AKT (S473), AKT, p-S6 (S235/236), S6, and p85 in sg-Ctrl and *RNF43*^659mut^ cells alone or with sg-*RNF43*-KO or RSPO1 (500 ng/ml) treatment. **C** Schematic representation of the quantitative proteomics workflow used to analyze RNF43 p.G659Vfs protein–protein interactions. Schematic created with BioRender.com. **D** Volcano plot illustrating the significance (*y*-axis) and magnitude (*x*-axis) of protein–protein interactions enriched in RNF43-p.G659Vfs*41 relative to RNF43-wildtype (WT) as detected by immunoprecipitation–mass spectrometry. **E** p85 expression in *RNF43*^659mut^ edited isogenic and sg_Ctrl CRC cells with and without MG132 treatment. **F** Ubiquitin immunoprecipitation assay assessing p85 ubiquitination in vector, RNF43_WT (WT), and RNF43-G659Vfs*41 (G659Vfs) expressing 293T cells. **G** Binding between p85 and RNF43-WT (WT), RNF43-G659Vfs*41 (G659Vfs) and RNF43-N terminal from 1aa to 659aa truncation (659_stop). **H** p85 expression in 293T cells transfected with RNF43-WT (WT), RNF43_G659Vfs (G659Vfs), RNF43_659_stop (659_stop), or RNF43_659ΔRING (ΔRING). Source data are provided as a Source Data file.

Overexpression of *RNF43*_p.G659fsV*41 in 293T, HCT116, and HT29 cells increased levels of p-AKT, p-S6, and p-4EBP1 (Supplementary Fig. 5). HT29 and LS513 *RNF43*^659mut^-edited single clones also showed increased expression levels of p-AKT, p-S6, and p-4EBP1 (Fig. 4A and Supplementary Fig. 6). Treatment with alpelisib and PF-04691502 specifically attenuated PI3K signaling in HT29-*RNF43*^659mut^ cells in a dose-dependent manner and in *RNF43*^659mut^ CDX models (Figs. 4A and 3E). *RNF43* knockout or RSPO1 treatment reversed the phosphorylation of p-AKT, p-S6 and p-4EBP1 in isogenic HT29 RNF43^659mut^ cells (Fig. 4B and Supplementary Fig. 6). In addition, *RNF43*^659mut^ knockout, RSPO1 treatment, or siRNA mediated AKT silencing reduced the increased cell growth conferred by *RNF43*^659mut^ (Supplementary Figs. 7 and 8). Importantly, in the Cancer Cell Line Encyclopedia and TCGA CRCs^23,24^, we also found that p-AKT and p-S6 levels were increased in *RNF43*^659mut^ cell lines and tumors, respectively (Supplementary Fig. 9a, b).

To understand the molecular link between RNF43^659mut^ and PI3K/AKT signaling, we sought to identify proteins that interact with RNF43^659mut^ by affinity proteomics, and that could mediate PI3K/AKT activation. V5-tag-RNF43_p.G659Vfs and V5-tag-RNF43_WT were overexpressed in 293T cells, and immunoprecipitated under non-denaturing conditions. The immunoprecipitated proteins were digested, labeled with tandem mass tag reagents (TMT) for quantification and analyzed by LC-tandem mass spectrometry (MS) (Fig. 4C). Interestingly, the PI3K regulating subunit p85 (also known as PIK3R1) emerged as a novel interacting partner of RNF43_p.G659Vfs but not RNF43_WT (Fig. 4D and Supplementary Data 2). Indeed, we showed reduced p85 levels in *RNF43*^659mut^ HT29 cells which were restored with RSPO1 supplementation or RNF43 knockout (Fig. 4B). p85 reduction was due to post-translational degradation and its expression was restored upon proteasome inhibition in *RNF43*^659mut^ HT29, LS513, and 293T, cells, but not upon lysosome inhibition in 293T cells (Supplementary Figs. 10 and 11a and Fig. 4E). In addition, p85 knockdown in HT29 and C10 cells showed increased p-AKT expression and led to increased colony formation in HT29 cells (Supplementary Fig. 11b, c).

We next sought to investigate whether p85 is ubiquitinated by RNF43^659mut^. We assessed p85 ubiquitination in cells expressing vector, RNF43_WT and RNF43_p.G659Vfs*41 by immunoprecipitating ubiquitinated proteins and observed increased p85 ubiquitination with RNF43_G659Vfs (Fig. 4F). Similar results were obtained with the reciprocal immunoprecipitation (Supplementary Fig. 12) and Tandem Ubiquitin Binding Entities (TUBEs) immunoprecipitation in isogenic *RNF43*^659mut^ HT29 cells (Supplementary Fig. 13). To further study the interaction between RNF43 and p85 we used RNF43-WT (WT), RNF43 from 1aa to 659aa (659_Stop), RNF43_G659Vfs (G659Vfs) and RNF43_659 fs containing a RING domain deletion (ΔRING)^25,26^. We observed that p85 binds with G659Vfs and 659_Stop but not the wildtype RNF43 (Fig. 4G). RNF43_G659Vfs and RNF43_659_Stop decreased p85 expression and this decrease was reversed by deletion of the RNF43_659fs RING domain, further supporting RNF43-mediated ubiquitination as the mechanism (Fig. 4H). To understand the structural basis of the RNF43_659 fs–p85 interaction, we computationally modeled^27,28^ RNF43 and observed that the C-terminus of the RNF43 model is negatively charged (residues 736–781), folding back to a positively charged region (residues 551–578), which could block binding of potential partners (Supplementary Fig. 14a). Thus, the truncated structure of RNF43^659mut^ may expose a positively charged site, allowing for increased binding and ubiquitination of p85 (Supplementary Fig. 14b). Overall, these results demonstrate that p85 interacts with RNF43^659mut^ and that ubiquitination and degradation of p85 leads to increased PI3K/AKT signaling in *RNF43*^659mut^ cells.

### RNF43^659mut^ modulates interferon activity

**Fig. 5:**
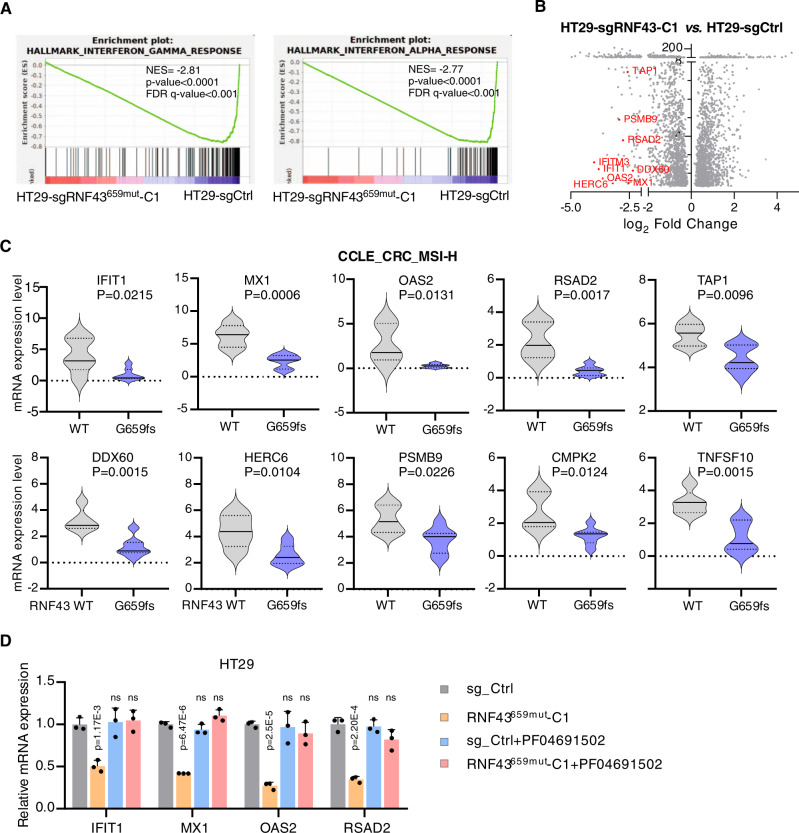
Modulation of interferon alpha and interferon gamma response genes in *RNF43*^659mut^ HT29 cells. **A** RNA-seq GSEA results in HT29_*RNF43*^659mut^ reveal decreased expression of interferon alpha and interferon gamma response genes. NES normalized enrichment score. **B** Volcano plot of differentially expressed genes in HT29_*RNF43*^659mut^ vs. HT29_sgCtrl. Interferon alpha or gamma response genes are displayed in red. **C** Differential expression of interferon alpha or gamma response genes in G659fs vs. WT MSI-H CRC cell lines in the Cancer Cell Line Encyclopedia (CCLE). Two-sided *t*-tests were used to compare RNF43_WT (*n* = 5) and RNF43_G659fs (*n* = 7) cells. **D** Comparison of interferon response genes between HT29_sgCtrl and HT29_*RNF43*^659mut^-C1 before and after treatment with PF-04691502. Data are the mean ± SD. Two-sided Student’s *t* test (*n* = 3 independent experiments in triplicate). Source data are provided as a Source Data file.

*RNF43*^659mut^ frequently occurs in CRCs with deficient DNA mismatch repair (dMMR)/high microsatellite instability (MSI-H) that are under immune pressure^16^, while the PI3K/AKT pathway is involved in immunomodulation and immunosurveillance escape in CRC^29^. Having found that RNF43^659mut^ activates the PI3K/AKT pathway, we sought to identify other genes that are modulated in *RNF43*^659mut^ and may contribute to immune evasion. We performed whole-transcriptome analysis (RNA-Seq) and Gene Set Enrichment Analysis (GSEA)^30^ in the *RNF43*^659mut^ and *RNF43*^WT^ isogenic HT29 cell lines. *RNF43*^659mut^ cells demonstrated features that negatively correlated with interferon-alpha and interferon-gamma response gene sets that contribute to inflammatory and innate immune responses, respectively (Fig. 5A, B and Supplementary Fig. 15). Consistently, in the TCGA Colorectal Adenocarcinoma dataset (*n* = 500 CRCs), the mRNA expression of interferon pathway genes were significantly lower in the samples with *RNF43*_p.G659fs mutation than those with wildtype *RNF43* (Fig. 5C). Lastly, treatment with PF-04691502 restored expression of interferon pathway genes in *RNF43*^659mut^ HT29 cells (Fig. 5D).

## Discussion

*RNF43* is a recurrently mutated gene in CRC and other malignancies, but the functional effects of its hot-spot variants warrants further investigation. Previous studies by our group and others have pointed at functional heterogeneity among *RNF43* mutations found in patients^31,32^. *RNF43* deleterious N-terminal domain mutations have been shown to activate Wnt signaling pathways, which in turn confers susceptibility to Wnt-ligand inhibitors such as PORCN inhibitors^13,15^. On the other hand, onco-*RNF43* mutations (504aa-568aa) lead to impaired Wnt receptor endocytosis and increased Wnt signaling but decreased susceptibility to PORCNi^32^. However, the role of C-terminal mutations of *RNF43* in Wnt signaling and oncogenesis remains controversial^33^. Two previous reports demonstrated that *RNF43*_p.G659fs mutations do not compromise *RNF43* activity, suggesting that it is a passenger event^18,19^. Nevertheless, the frequency of the *RNF43*_p.G659fs truncation mutant was shown to be significantly greater than predicted by chance in colorectal and endometrial tumors indicating positive selection^16^.

In this work, we demonstrated that *RNF43*_p.G659fs mutations have tumorigenic properties independent of Wnt signaling. In agreement with our findings, previous research showed that treatment with PORCN inhibitors had no effect on CRC organoids with 659fs mutations^15^. In order to evaluate specific therapeutics against tumors harboring the *RNF43*_p.G659fs mutation, we utilized the Broad Institute’s drug repurposing library^21^ and identified a significant enrichment in PI3K/mTOR inhibitors such as PF-04691502 and alpelisib. PF-04691502 is a dual PI3K/mTOR inhibitor in clinical trials, while alpelisib is an FDA-approved drug for the treatment of advanced breast cancer^34^. In this work, we showed that alpelisib and PF-04691502 inhibit the growth of preclinical tumor models harboring the *RNF43*_p.G659fs mutation. We demonstrated that this mutation activates PI3K in vitro and in vivo and showed that this activation occurs through degradation of PI3K regulatory subunit p85. A caveat of these studies is that since, to the best of our knowledge, there are no patient-derived isogenic cells for the *RNF43* p.G659fs mutation, we used CRISPR-Cas9 genomic editing to generate isogenic models.

p85 has previously been found to inhibit PI3K/AKT signaling through the inhibition of p110^35^, while knockdown of p85 promotes oncogenic activation of PI3K/AKT effector signaling^36–39^. Similarly, inactivating mutations of p85 increased p-AKT levels, while wildtype p85 had no effect on p-AKT levels^40^. We find that RNF43_p.G659fs removes p85 inhibition of PI3K/AKT signaling through binding, ubiquitinating and degrading p85, and that RNF43_p.G659fs-induced activation of PI3K/AKT signaling can be reversed by PI3K/mTOR inhibitors.

*RNF43*_p.G659fs mutations are predominantly present in MSI‐H CRCs, which are characterized by increased immune cell infiltration and have ~40–50% response rate to immune checkpoint inhibition^34^. We showed that *RNF43*^659mut^ leads to decreased expression of interferon-alpha and gamma response genes in vitro and in TCGA CRCs. This diminished activity was restored in CRC cells through PI3K/mTOR inhibition. Given this role of *RNF43_*p.G659fs in immunomodulation, it is intriguing to speculate about its potential effect on immune checkpoint blockade resistance and the possibility of combination immunotherapy trials with PIK3/mTOR inhibitors. Additional studies are needed, however, to further investigate the role of RNF43_p.G659fs in immunotherapy failure in MSI-H CRC.

Overall, our findings provide new insights into the role of *RNF43*_p.G659fs in tumor development and suggest therapeutic uses for PI3K/mTOR inhibitors in patients whose tumors harbor this mutation.

## Methods

### Cell culture

HT29 (RRID:CVCL_0320), HCT116 (RRID:CVCL_0291), LS513 (RRID:CVCL_1386), HEK293T (RRID:CVCL_QW54), L-Wnt3a (RRID:CVCL_0635) cell lines were obtained from the American Type Culture Collection, 293T-HA-RspoI-Fc (RRID:CVCL_RU08) was obtained from Trevigen (# 3710-001-K) and C10 cell line (ECACC Cat# 12022901, RRID:CVCL_5245) was obtained from the European Collection of Authenticated Cell Cultures (ECACC). All cell lines were cultured under standard conditions recommended by the manufacturer supplemented with 10% FBS (Sigma-Aldrich, #F2442) and 1% penicillin/streptomycin (Thermo Fisher Scientific, #BW17603E) and kept at 37 °C in a humidified atmosphere with 5% CO_2_. All cell lines were authenticated by validating short tandem repeat fingerprinting and were free of mycoplasma contamination.

### Organoid culture

Colon tissues in this study were obtained and used for research purposes under protocols approved by Dana-Farber Cancer Institute’s IRB with informed consent from patients. For establishment of CRC patient-derived organoids, tumor tissues were obtained from patient-derived *RNF43*_G659fs xenograft models. For establishment of normal patient-derived colorectal organoids, tissue was obtained from surgical specimens. The organoid samples were dissociated and embedded in Matrigel (Corning #356231). After Matrigel solidification for 10 min at 37 °C, cells were cultured in human intestinal organoid medium composed of IntestiCult™ Organoid Growth Medium (STEMCELL Technologies, #06010) with 100 U/ml penicillin-streptomycin (Thermo Fisher Scientific, #15140122) and supplemented with Y-27632 for 48 h after establishment or passage. Confluent organoids were passaged by incubating in Gentle Cell Dissociation Reagent (STEMCELL Technologies, #100-0485). Organoids were resuspended in a 1:1 mixture of Matrigel and basal media composed of DMEM/F12 (Thermo Fisher Scientific, #11320033), 10 mM HEPES (Life Technologies, #15630080), 2 mM GlutaMax (Life Technologies, #35050061), and 100 U/ml penicillin-streptomycin.

### Human specimens

Human Colonic tissues and clinical data were obtained from CRC patients at Dana-Farber Cancer Institute following informed consent and IRB-approval. Clinical data corresponding to patient-derived specimens is listed in Supplementary Table 2. TCGA datasets (lluminaHiSeq_RNASeqV2data and DNA mutations using Affymetrix Genome-Wide Human SNP 6.0 array data) were obtained from the cBioPortal (https://www.cbioportal.org/). TCGA proteomic/Reverse phase protein array data were obtained from The Cancer Proteome Atlas (http://tcpaportal.org/tcpa/).

### Plasmids and transfection

For overexpression experiments, *RNF43* wildtype open-reading frame constructs (ORFs) were obtained from the human ORFeome library version (http://horfdb.dfci.harvard.edu) and used as templates for site-directed mutagenesis to generate *RNF43*_G659fs*41 mutated cDNAs in the pDONR223 Gateway entry vector followed by Sanger sequencing verification. Ectopic expression of V5-tagged *RNF43* wildtype, G659fs*41 and indicated truncated fragments were cloned into the LentiV_Puro expression vector (gifted from Christopher Vakoc; RRID:Addgene_111886) with the HIFI DNA assembly kit (New England Biolabs #E2621S) according to manufacturer specifications. The RNF43_659fsΔRING plasmid was constructed by VectorBuilder (www.vectorbuilder.com). CRISPR-Cas9 genome editing, single guide RNAs were designed to target DNA sequences corresponding to amino acid residues 117 (sgRNA_*RNF43*^117*mut*^: 5′-TAGCCAGTGACAGGCAGGGG-3′) and 659 (sgRNA_*RNF43*^659mut^: 5′-CCACAGAGGAAAAGGCGGGG-3′) of *RNF43* using GPP sgRNA Designer (https://portals.broadinstitute.org/gpp/public/analysis-tools/sgrna-design) and introduced into the lentiGuide_Puro backbone (gift from Feng Zhang; RRID:Addgene_52963) following Lenti_Cas9-2A-Blast (RRID:Addgene_73310) transfection. Additionally, the TCF/LEF sequence was PCR amplified and inserted into a pGL4.15 vector (Progema, #E6701) via HIFI DNA assembly to create a TOP luciferase reporter vector. Plasmid constructs were sequenced and confirmed by Sanger sequencing (Quintara Bioscience). All primers for plasmid construction and sgRNA target sequences are listed in Supplementary Table 3.

Lentivirus was produced in HEK293T cells as per the low throughput viral production protocol on the shRNA/sgRNA/ORF Low Throughput Viral Production Consortium Portal (see https://portals.broadinstitute.org/gpp/public/). Briefly, the HEK293T cells were plated at 70% confluence in 6-well plates 24 h prior to transduction. Lentivirus was produced by transfecting HEK293T cells with previously described plasmids as well as psPAX2 (RRID: Addgene_12260) and VSV-G (RRID: Addgene_8454) packaging plasmids. Stable transfection was performed in indicated cell lines by spin-infection lentivirus transduction and 48 h incubation in the presence of 5 μg/ml polybrene prior to the addition of puromycin (1 ug/ml) selection for 7 days. For transient transfection of plasmids or oligonucleotides into most mammalian cell lines, Lipofectamine LTX reagent (Thermo Fisher Scientific, #15338100) was used following the manufacturer instructions while HEK293T cell transfection was performed with the Calphos mammalian transfection kit (Takara Bio, #631312) as per manufacturer instructions.

### CRISPR/Cas9 genome editing

To generate isogenic *RNF43* mutant cell lines, cells were transduced with Lenti_Cas9-2A-Blast and an indicated Lenti-Guide-puro construct containing the sgRNA inserts. Blasticidin (2 ug/ml) and puromycin (1 µg/ml) selection treatments were applied for 7 days. To confirm efficient CRISPR cutting at the target loci, gDNA was extracted using QIAamp DNA mini kit (Qiagen, #51104) and PCR primers were designed flanking the sgRNA cut site by 100–150 base pairs on either side. DNA from the bulk resistant population of cells transduced with sgRNAs targeting *RNF43* 117 and *RNF43* 659 was amplified with the primers to yield an amplicon roughly 200–300 bp in length using Q5 High-Fidelity 2X Master Mix (New England Biolabs, #M0492S) and confirmed by next generation sequencing to determine editing efficiency (Massachusetts General Hospital DNA Core). Mutation efficiency was assessed as the percentage of reads containing a frameshift caused by an indel compared to the total read count (Supplementary Fig. 1a). Single colonies were selected by serial dilution and screened for frameshift mutations by Sanger sequencing of the PCR products generated using the primers that flanked the targeted site (Supplementary Fig. 1b). Editing efficiency was assessed by determining the percentage of sequences with frameshift mutations compared to the total number of polymers sequenced. The gDNA PCR primer sequences corresponding to the regions targeted by indicated sgRNAs are shown in Supplementary Table 4.

### Colony formation assays

Cells (1000 cells per well) were seeded in 12-well plates for 14 days. Cells were fixed with 100% methanol and then stained with 0.5% crystal violet for 5 min. Plates were imaged with an Epson Workforce GT-1500 scanner (Epson, B11B190011). Viable cellular colonies larger than the indicated diameter were counted. Colony formations assays were performed in triplicate.

### Cell and organoid viability and treatment assays

Cell viability was assessed using the CellTiter-Glo Luminescent cell viability assay (Promega #G9242) according to the manufacturer’s protocol and measured on a Spectramax M5 microplate luminometer (Molecular Devices). For the cell proliferation experiment, cells were plated (1000 cells per well) in 96 well plates and assayed daily for 6 days to determine cell proliferation. Cell treatments were conducted with alpelisib (Selleck #S2814), PF-04961502 (Selleck #S2743), Trametinib (Selleck #2673), Nutlin-3 (Selleck #8059), XAV939 (Santa Cruz Biotechnology #SC-296704), LGK974 (Selleck #S7143), IWP2 (Selleck #7085), PKI-179 (EMDBio #526561), Torin-1 (Selleck #S2827), and Torin-2 (Tocris #4248) with 7-point 2-fold dilution series for 96 h before the viability was assayed. For the organoid drug treatment experiments, organoids were dissociated to single cells (1000/well) and resuspended in Wnt-ligand free organoid growth media containing 10% Matrigel. Organoid viability was assessed using 3D CellTiter-Glo (Promega #G9681) after 7 days of drug treatment. Each assay was measured in triplicate using at least three technical replicates (two for the normal organoids) and luminescent output was normalized to cells treated with DMSO. The Linear regression analysis and dose-response inhibition (Ln(inhibitor) vs. normalized response—variable slope) or (Ln(inhibitor) vs. normalized response) were calculated by GraphPad PRISM (GraphPad).

### Dual-luciferase reporter assay

Cells were seeded in triplicate 1 day prior to transfection. In total, 50 ng of the previously assembled TOP/Wnt luciferase reporter vector or M50 Super TOPFlash (gift from Dr. Randall Moon; Addgene, #12456) were transfected into cells alongside 1 ng of pRL-TK Renilla plasmid (Promega, #E2241) by using lipofectamine 3000 (Invitrogen, #L3000001) according to the manufacturer specifications. Forty-eight hours post-transfection, firefly and Renilla luciferase activities were measured by dual-luciferase assay kit (Promega, #E1910) on a Spectramax M5 (Molecular Devices) according to the manufacturer’s instructions. The firefly luciferase activity for each sample was normalized to the Renilla luciferase activity to account for transfection efficiency. Each assay was measured in triplicate using at least two technical replicates.

### Small molecule compound profiling

The Broad Institute Repurposing Library screen was performed using 5363 small molecules arrayed in 384-well plates at a final concentration of 5 uM. Assay Ready Plates (Corning Cat# 3765) were generated by dispensing 10 mM DMSO compound stocks using the Labcyte Echo acoustic liquid handler. HT29-sgCtrl and HT29-*RNF43*^659mut^ isogenic cell lines were seeded in duplicate at a density of 2500 cells per well in 50 ul culture medium supplemented with 10% FBS. Assay plates were incubated for 96 h and cell viability was measured using CellTiter-Glo per manufacturer recommendation. Quality control measures were met for screening plates with a *Z*-factor score >0.5 comparing negative (DMSO) and positive control compound MG132 (a proteasome inhibitor toxic to most cell lines at 1 µM) and %CV < 15%. Compounds which reliably inhibited cell growth (<10% difference between replicates; *z*-score < −3) and exhibited at least 5% more growth inhibition in HT29-*RNF43*^659mut^ than in HT29-*RNF43*_sgCtrl were considered primary hits. These primary hits were used to generate a secondary screen consisting of an 8-point 2-fold dilution series to generate a concentration-response curve and assess drug potency and sensitivity.

### Mouse experiments

All animal experiments and study protocols were reviewed and approved by the Dana-Farber Cancer Institute’s Animal Care and Usage Committee (protocol 20-013), in compliance with the Animal Welfare Act and the Office of Laboratory Welfare (OLAW) of the National Institutes of Health. Laboratory mice are housed in solid-bottom, polysulfone 75 sq. in. microisolator cages. The cages are used in conjunction with the Optimice® rack systems with integrated automatic watering. Temperature and humidity in the rodent facilities are controlled at 72 ± 2 °F and a target range of 35–55% relative humidity. A standard photoperiod of 12 h light/12 h dark is controlled by an automated system. For the cell-derived xenograft assay, 2 × 10^6^ cells of HT29-sgCtrl or HT29-*RNF43*^659mut^ in 100 µl of a 50% PBS/50% Matrigel mixture were injected subcutaneously into flanks bilaterally in Taconic NCr-Nude (CrTac:NCr-Foxn1nu) female mice at 7 weeks of age. When tumors reached ~150 mm^3^ after 7 day of tumor cells inoculation, mice (*n* = 5/group) were randomized into vehicle control (5%DMSO + 40%PEG300 + 5%Tween 80 + 50%ddH2O) and PF-04961502 treatment (50 mg/kg/day by oral gavage) groups for 5 days on and 2 days off. On day 28 of treatment, mice were euthanized. The endpoints for mice were tumor volume exceeding 2000 mm^3^, interference with basic/vital bodily functions or persistent ulceration. At no point did any mice exceed maximal tumor burden. Blinding and randomization to the treatment arm was performed. When endpoints were reached, tumors were removed from mice, washed, and stored overnight in 4% paraformaldehyde. Histological analysis and immunostaining of paraffin-embedded tumor-samples was then performed by iHisto Inc. Anti-Ki67 (Abcam #15580), anti-p-AKT (Cell Signaling Technologies #4060T) were used for immunohistochemistry analysis, while TUNEL staining was provided and performed by iHisto Inc.

### Western blot analysis

Whole cell extracts for western blotting were obtained by applying RIPA buffer (Sigma-Aldrich, #R0278) supplemented with protease inhibitor (Cell Signaling Technology, #5872S) onto a cell monolayer and incubating on ice for 5 min. Following centrifugation (>16,000 × *g*) for 10 min), protein concentrations were quantitated using the Pierce BCA Protein Assay Kit (Thermo Fisher Scientific, #23225). Equal amounts of protein were resolved and transferred according to manufacturer specifications, followed by overnight incubation at 4 °C with the primary antibodies listed in Supplementary Table 5. Chameleon Duo Prestained Protein Ladder (Licor, #928-60000) or PageRuler Prestained Protein Ladder (Thermo Fisher Scientific, #26616) were used as a molecular weight marker. Densitometry analysis of each blot was quantitated using ImageJ. Uncropped immunoblots are provided in Source Data.

### Proteasome and lysosome inhibition assays

To detect p85 expression in LS513 and HT29 cells, cells were incubated with 10 μM MG132 (Sigma-Aldrich) for 6 h before analysis. Additionally, HEK293T expressing RNF43_G659Vfs were treated with MG132 (Selleck, #S2619), Aloxistatin (E64d) (Selleck, #S7393) or NH_4_Cl (Sigma-Aldrich, #S7393). Then, cells were lysed in RIPA buffer (50 mM Tris-HCl pH 7.4, 150 mM NaCl, 1% NP-40, 0.1% SDS, 0.5% sodium deoxycholate, 2 mM Na3VO4, 20 mM NaF, 1 mM PMSF and complete protease inhibitor cocktail) and subjected to immunoblotting. Expression levels of p85 were quantified and normalized to beta-actin using ImageJ.

### Cycloheximide chase assay

HEK293T cells were transfected with RNF43 (wildtype or G659Vfs mutant) for 24 h and further incubated with protein synthesis inhibitor 5 μM Cycloheximide (CHX, Sigma-Aldrich, #C104450) for the indicated amount of time before analysis.

### Co-immunoprecipitation assays

HEK293T cells were transiently transfected with purified V5-tagged-*RNF43*-WT or -*RNF43*_G659Vfs*41 plasmids using the Calphos Mammalian Transfection kit (Takara Bio, #631312) and incubated for 24–72 h before protein is harvested. Cells were washed with cold PBS and lysed with IP lysis buffer (Thermo Fisher Scientific, #87787) or NP40 cell lysis buffer (Thermo Fisher Scientific, #FNN0021) containing Halt Protease Inhibitor Single-use Cocktail (Thermo Fisher Scientific, #PI78430) and incubated 10 min on ice. After pelleting the cell debris by centrifugation at 16,000 × *g* for 10 min at 4 °C, supernatants (cell extracts) were incubated with 2 ug of anti-ubiquitin antibody FK2 (Sigma-Aldrich, #04-263) or 1 ug anti-V5 antibody and 10 ul each of Protein A/G Dynabeads (Thermo Fisher Scientific, #88802) at 4 °C overnight while rotating. After four times extensive washing with IP buffer, the immunoprecipitated bead conjugates were heated in 2 × NuPAGE LDS Sample Buffer (Thermo Fisher Scientific, #NP0007) supplemented with 20 mmol/l DTT at 70 °C for 10 min and subjected to NUPAGE 4–12% BT GEL (Thermo Fisher Scientific, #NP032B), followed by immunoblotting probed with an antibody against P85. TUBE immunoprecipitations were carried out similarly; cells were treated with MG132 and 1 mg of HT29 or HT29-*RNF43*^659mut^ cell lysate was loaded onto 20 uL TUBE1 agarose (LifeSensors, #UM401). Antibodies are listed in Supplementary Table 5.

### Proteomic analysis

Sample were processed for quantitative MS assays following anti-V5 immunoprecipitation by on-bead digestion (add: PMID 33139955). Briefly, for protein interaction analyses, samples were reduced, alkylated, and digested on-bead with trypsin as previously described (add: PMID 33139955). Desalted peptides were labeled with TMT (8-plex) reagents (Thermo Fisher Scientific), combined and analyzed by online nanoflow liquid chromatography-tandem MS using a Q Exactive Plus mass spectrometer (Thermo Fisher Scientific) coupled online to a Proxeon Easy-nLC 1200 (Thermo Fisher Scientific) using a 260 min method. Briefly, the Q Exactive plus MS was operated in the data-dependent mode, acquiring HCD-MS/MS scans at a resolution of 35,000 on the top-12 most abundant precursor ions in each full MS scan (70,000 resolution). The automatic gain control target was set to 3 × 10^6^ ions for MS1 and 5 × 10^4^ for MS2, and the maximum ion time was set to 120 ms for MS2. The collision energy was set to 28, peptide matching was set to “preferred,” isotope exclusion was enabled, and dynamic exclusion time was set to 20 s. All data were analyzed using Spectrum Mill software package. Spectra from the same precursor or within a retention time window of ±60 s and an *m/z* range of ±1.4 were merged. Spectra were filtered to include only those with a precursor mass range of 750 to 6000 Da and a sequence tag length greater than 0. MS/MS searching was performed against a human UniProt database. Digestion enzyme conditions were set to “Trypsin allow P” for the search, allowing up to 4 missed cleavages within a matched peptide. Fixed modifications were carbamidomethylation of cysteine and TMT10 on the N-terminus and internal lysine. Variable modifications were oxidized methionine and acetylation of the protein N-terminus. Peptide-level matches were validated below a 1.0% FDR threshold and within a precursor charge range of 2–6. A second round of validation was performed at the protein level, requiring a minimum protein score of 0. Protein data were filtered to consider only proteins with two or more unique peptides and median normalized. Data were median normalized and subjected to a 1-sample moderated *t-*test using an internal R-Shiny package based in the limma R library. Correction for multiple testing was performed using the Benjamini–Hochberg FDR method. Proteins were selected based on increased binding in *RNF43*_G659Vfs*41 compared to *RNF43*_WT and a significant *p* value <0.05.

### RT-qPCR and RNA-sequencing

Total cellular RNA was isolated using the RNeasy plus mini kit (Qiagen, #74104) and subsequently underwent reverse transcription using the iScript cDNA synthesis kit (Bio-Rad Laboratories, #1708891) according to manufacturer instructions. RT-PCR was performed with Bio-Rad CFX96 qPCR System using PowerUp SYBR Green cDNA kit (Thermo Fisher Scientific, #A25741). GAPDH was used as an internal control for normalization, and the relative expression level was calculated by the comparative CT (ΔΔCT) method. Each assay was measured in triplicate using at least two technical replicates. Primer sequences are listed in Supplementary Table 6.

RNA-Sequencing of the isogenic cell lines was performed by Novogene Corporation Inc Service using the Illumina NovaSeq 6000 with an average read depth of at least 20 million reads per replicate. In total, 150 bp paired-end sequencing reads were aligned to the human genome reference build hg19 using STAR v2.5. Differential expression analysis was performed using the DESeq2 R package (2_1.6.3) and thresholds for significant expression gene were defined as *q* < 0.05 and |log2 fold-change| > 0.5. Pathway enrichment analysis was performed using GSEA 2.0.9 software with the hallmark gene sets database (https://www.gsea-msigdb.org/gsea/msigdb/collections.jsp). Gene sets with a *q* < 0.25 false discovery rate and *p* value <0.05 were selected for significant pathway enrichment. All *p* values were adjusted for multiple testing using the Benjamini–Hochberg method (false discovery rate).

### Statistics and reproducibility

Statistical analyses were performed using Graphpad Prism software (version 9.0.1 (128) (San Diego, CA). Student’s *t* test was used to determine the statistical differences between two groups. Experiments were performed in triplicate using at least three independants replicates unless otherwise indicated. All experiments were presented as the means ± standard deviation and all tests are two-sided.

### Reporting summary

Further information on research design is available in the Nature Research Reporting Summary linked to this article.

## Supplementary information

Supplementary Information

Description of Additional Supplementary Files

Supplementary Data 1

Supplementary Data 2

Reporting Summary

## Data Availability

Data supporting the findings of this study are available within the article, the Supplementary Information files, and the Source Data file. The Cancer Genome Atlas (TCGA, PanCancer Atlas) and Cancer Cell Line Encyclopedia (Broad, 2019) datasets were accessed via CBioPortal (https://www.cbioportal.org/). Uniprot human database (https://www.uniprot.org/) was used for identification of proteins by mass spectrometry. Proteomic GSEA experiments were performed using GSEA 2.0.9 software with the hallmark gene sets database (https://www.gsea-msigdb.org/gsea/msigdb/collections.jsp). The RNA-seq data for this study have been deposited to the GEO public dataset under the series GSE179464. The original mass spectra and the protein sequence database used for searches have been deposited in the public proteomics repository MassIVE (http://massive.ucsd.edu) and are accessible at under accession code MSV000089343. Source data are provided with this paper.

## Source data

Source Data
