# Supplementary material for: RNF43 G659fs is an oncogenic colorectal cancer mutation and sensitizes tumor cells to PI3K/mTOR inhibition
Source: Nat Commun. 2022 Jun 8;13:3181. doi: 10.1038/s41467-022-30794-7 (PMC9177965; doi:10.1038/s41467-022-30794-7)
Supplement: Supplementary file 1 — Supplementary Information [file 41467_2022_30794_MOESM1_ESM.pdf]

Supplementary Fig. 1 | Genome-Editing of *RNF43* in CRC cell lines

A

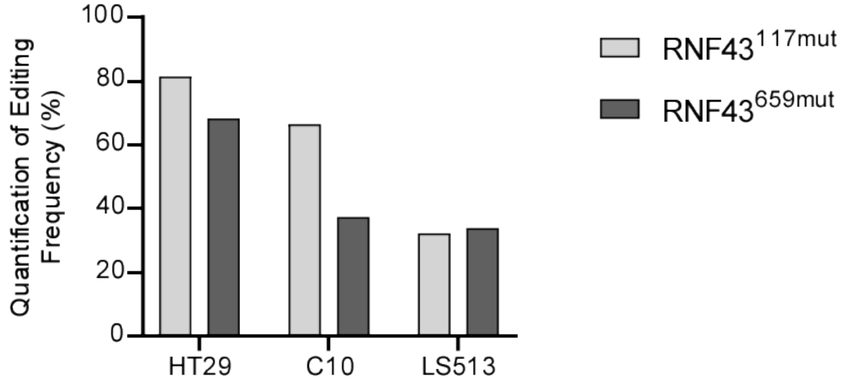

B

```
                                     657  658  659  660  661
WT          CGACACCCACAGAGGAAAAGGCGGGGGGGTCCCTCCGAGCCCACCCCTGGCTCT
             R  H  P  Q  R  K  R  R  G  G  P  S  E  P  T  P  G  S
Sg-Ctrl     CGACACCCACAGAGGAAAAGGCGGGGGGGTCCCTCCGAGCCCACCCCTGGCTCT
C1-C5        R  H  P  Q  R  K  R  R  G  G  P  S  E  P  T  P  G  S
HT29-C1     CGACACCCACAGAGGA----------------------GCCCACCCCTGGCTCT (-22)
             R  H  P  Q  R                          S  P  P  L  A
HT29-C2     CGACACCC-------------------------TCCGAGCCCACCCCTGGCTCT (-25)
             R  H  P                              P  S  P  P  L  A
HT29-C3     CGACACCCACAGAGGAAAAGG--GGGGGGTCCCTCCGAGCCCACCC-TGGCTCT (-3)
             R  H  P  Q  R  K  R   G  G  S  L  R  A  H  P  G  S
HT29-C4     CGACACCCACAGAGGAA--GG--GGGGGGTCCCTCCGAGCCCACCCC-GGCTCT (-5)
             R  H  P  Q  R  K   G    G  V  P  P  S  P  P  R   L
HT29-C5     CGACACCCACAGAGG------CGGGGGG-TCCCTCCGAGCCCACCCCTGGCTCT (-7)
             R  H  P  Q  R         R  G   V P  P  S  P  P  L  A  S
HT29-C6     CGACACCCACAGAGGAAAAGGCGGGGGGGGTCCCTCCGAGCCCACCCCTGGCTC (+1)
             R  H  P  Q  R  K  R  R  G  G  S  L  R  A  H  P  W  L
HT29-C7     CGACACCCACAGAGGAAAAGAGGCGGGGGGGTCCCTCCGAGCCCACCCCTGGCT (+2)
             R  H  P  Q  R  K  R  R  G  G  S  L  R  A  H  P  W  L
LS513-C8    CGACA----------------------------------CCCACCCCTGGCTCT (-34)
             R  H                                     P  P  L  A
LS513-C9    CGACACCCACAGAGGAAAAGGCGG-----TCCCTCCGAGCCCACCCCTGGCTCT (-5)
             R  H  P  Q  R  K  R  R        S  L  R  A  H  P  W  L
```

C

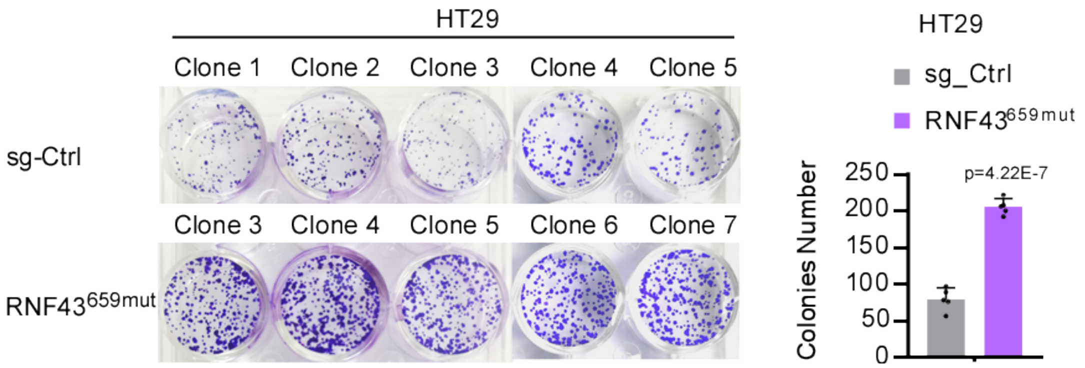

A, Quantification of CRISPR/Cas9 *RNF43*_G659fs and *RNF43*_R117fs editing in HT29, C10 and LS513 cells. **B,** Next generation sequencing of isogenic clones displaying *RNF43*_G659fs editing in HT29 and LS513 cells. Values in parentheses represent the total number of absent or additional nucleotides. **C**, Representative colony formation assays of 5 HT29-sg-Ctrl and HT29-$RNF43^{659mut}$ isogenic clones (C3 to C7) in the absence of Wnt ligand and quantification of average number of sg_Ctrl and $RNF43^{659mut}$ colonies larger than 10 µm. Data are the mean ± SD. Student's t-test (*n* = 3 independent experiments in triplicate). Source data are provided as a Source Data file.

Supplementary Fig. 2 | Wnt pathway protein expression in HT29-*RNF43*^{659mut} cells

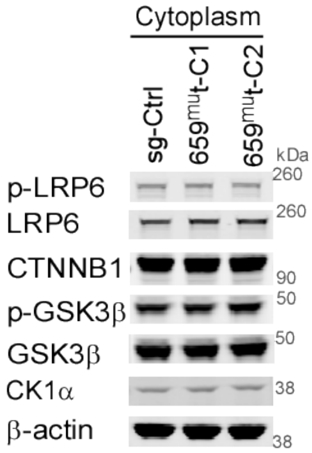

Western blot analysis of Wnt pathway proteins p-LRP6, LRP6, CTNNB1, p-GSK3β, GSK3β, CK1α and β-actin loading control in HT29-sg-Ctrl and two isogenic HT29-*RNF43*^{659mut} cell clones. Source data are provided as a Source Data file

Supplementary Fig. 3 | Sensitivity of *RNF43*^{659mut} to mTOR/PI3K inhibitors

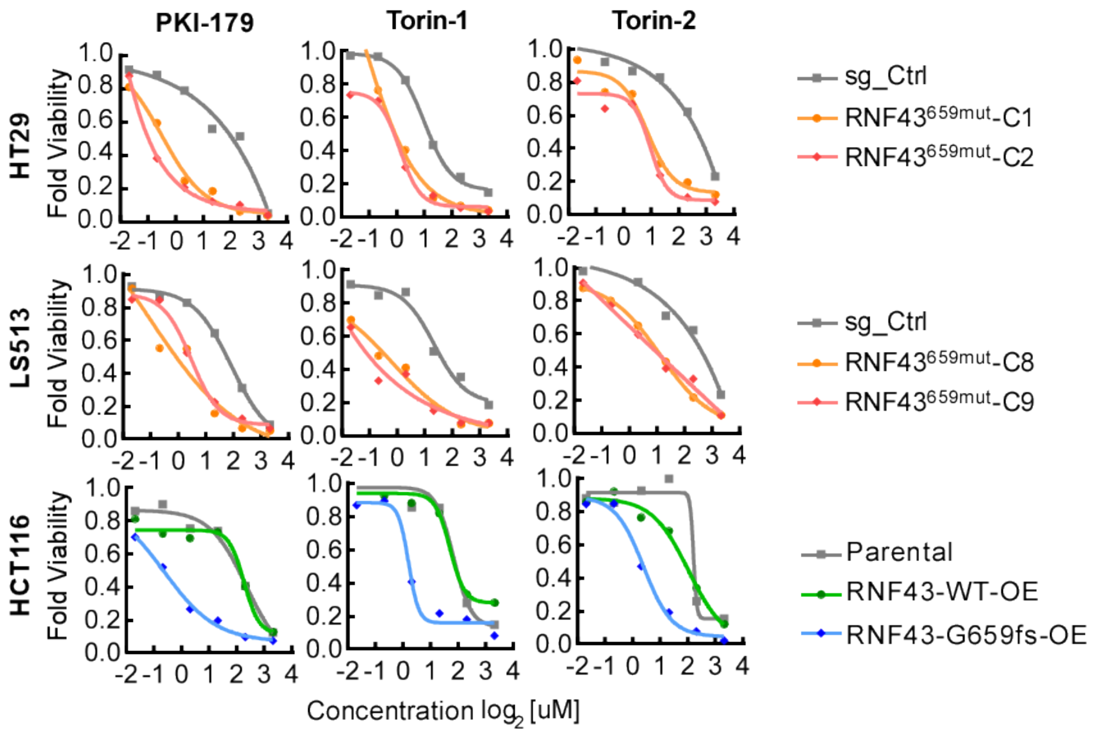

PKI-179, Torin-1 and Torin-2 dose-response curves in either *RNF43*^{659mut} edited cells (HT29, LS513) or ectopically expressing *RNF43*_G659Vfs*41 cells (HCT116) compared to controls. Experiments were performed in triplicate. Source data are provided as a Source Data file.

Supplementary Fig. 4 | PI3Ki sensitivity in TCGA CRCs

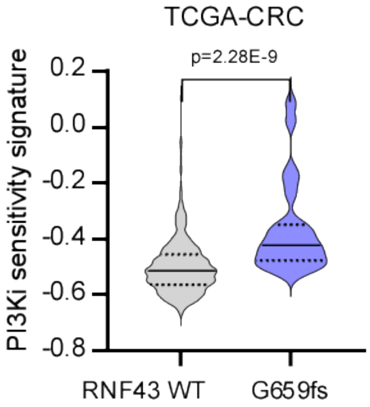

PI3K inhibitor sensitivity transcriptional signatures in RNF43_WT and RNF43_G659fs TCGA CRCs. Two-sided Student’s t-test. Source data are provided as a Source Data file.

Supplementary Fig. 5 | PI3K/mTOR pathway expression in WT and *RNF43*_G659fs overexpressing cells

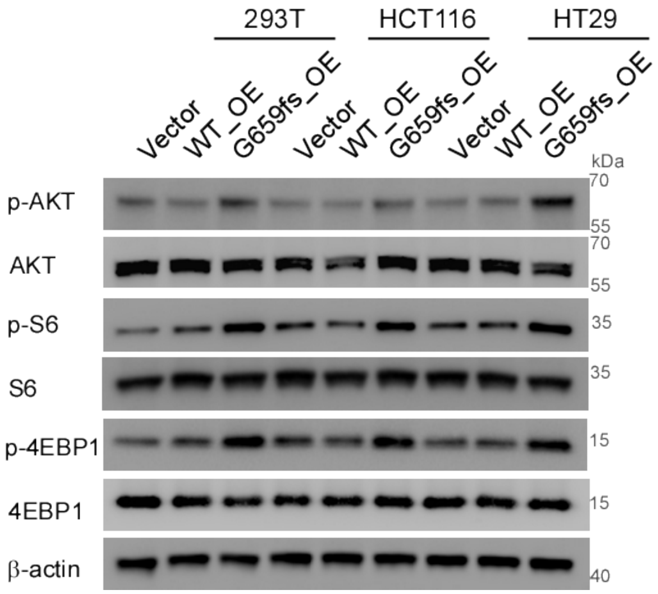

Western blot analysis of PI3K/mTOR pathway proteins p-AKT (S473), AKT, p-S6 (S235/236), S6, p-4EBP1, 4EBP1 in 293T, HCT116 and HT29 cell lines overexpressing empty vector (Vector), *RNF43*^{WT} (WT_OE) or *RNF43*_p.G659Vfs (G659fs_OE). β-actin shown as loading control. Source data are provided as a Source Data file.

Supplementary Fig. 6 | PI3K/mTOR pathway expression in isogenic HT29 clones

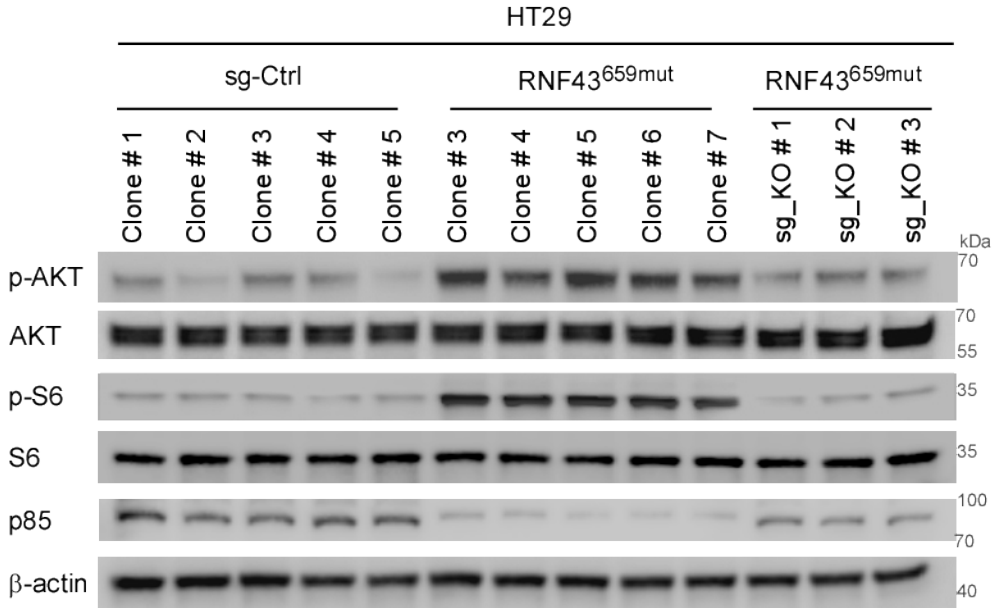

Western blot analysis of PI3K/mTOR pathway proteins p-AKT (S473), AKT, p-S6 (S235/236), S6, and p85 in isogenic HT29 sg-Ctrl, *RNF43*^{659mut} and *RNF43*^{659mut}-KO cells. Source data are provided as a Source Data file.

Supplementary Fig. 7 | Cell proliferation with *RNF43*^{659mut} knockout or inhibition

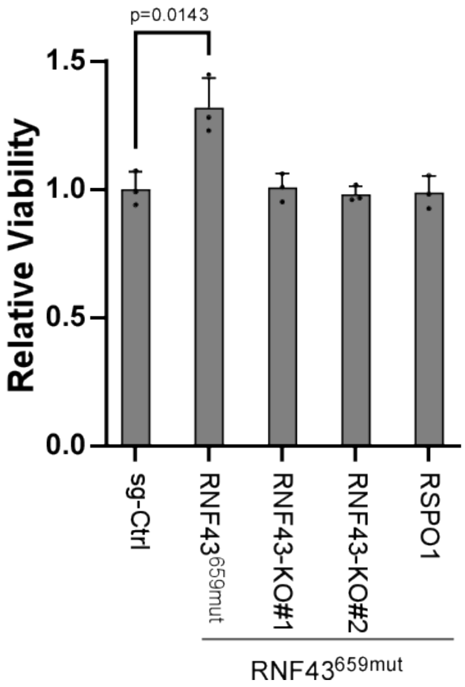

Cell Titer-Glo luminescence assay in sg-Ctrl and HT29 *RNF43*^{659mut}-C1 cells alone or with sg-RNF43-KO or RSPO1 (500 ng/mL) treatment. Data are the mean ± SD. Student's t-test (*n* = 3 independent experiments in triplicate). Source data are provided as a Source Data file.

Supplementary Fig. 8 | AKT knockdown in *RNF43*^{659mut} edited CRC cells

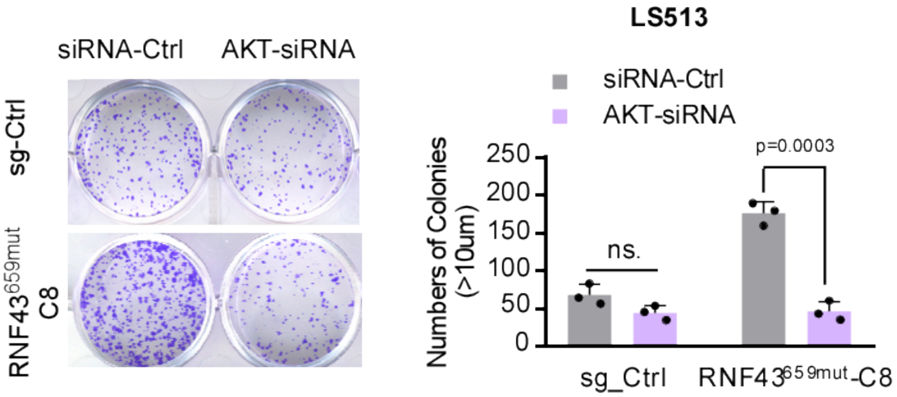

Representative colony formation assay (left) and quantification of number of colonies (right) after transfection of an AKT-siRNA on LS513 *RNF43*^{659mut}-C8 edited and sg-Ctrl isogenic cells. Data are the mean ± SD. Student's t-test (*n* = 3 independent experiments in triplicate). Source data are provided as a Source Data file.

Supplementary Fig. 9 | p-S6 and p-AKT expression in TCGA and CCLE CRCs

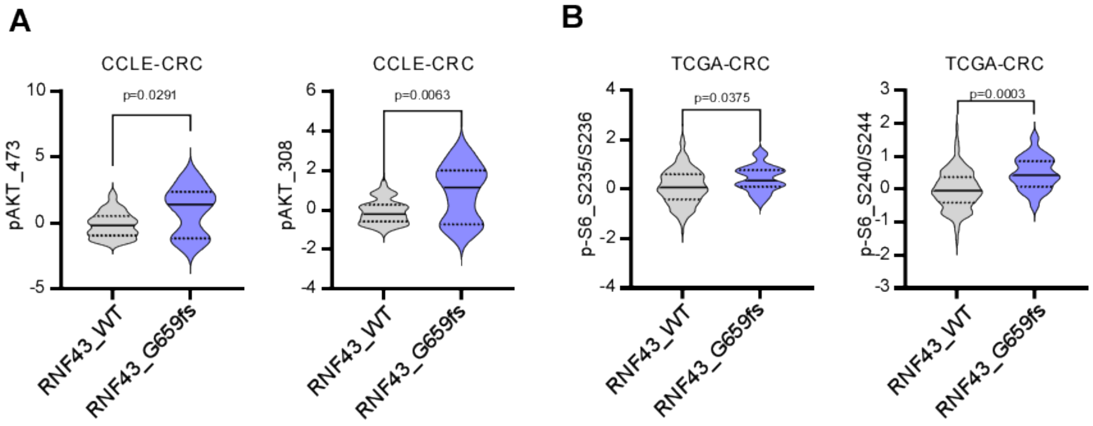

A, p- AKT_473 (left) and p-AKT_308 (right) expression in Cancer Cell Line Encyclopedia colorectal cancer (CCLE CRC) cell lines. Two-sided Student's t-test used to compare RNF43_WT (n=40), RNF43_G659fs (n=6). **B,** p-S6 S235/S236 (left) and p-S6_S240/S244 (right) expression in The Cancer Genome Atlas colorectal cancers (TCGA CRCs). Two-sided Student's t-test used to compare RNF43_WT (n=276), RNF43_G659fs (n=21). Source data are provided as a Source Data file.

Supplementary Fig. 10 | RNF43–G659Vfs reduces p85 post translational stability

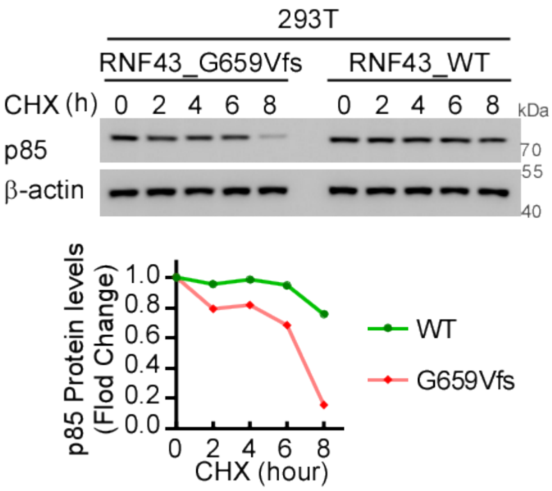

Western blot analysis (above) and quantification (below) of p85 expression following cycloheximide (CHX) treatment of 293T cells expressing RNF43_WT or RNF43_G659Vfs. Source data are provided as a Source Data file.

Supplementary Fig. 11 | p85 expression in WT and *RNF43*_G659fs cells and p85 knockdown impact on colony formation

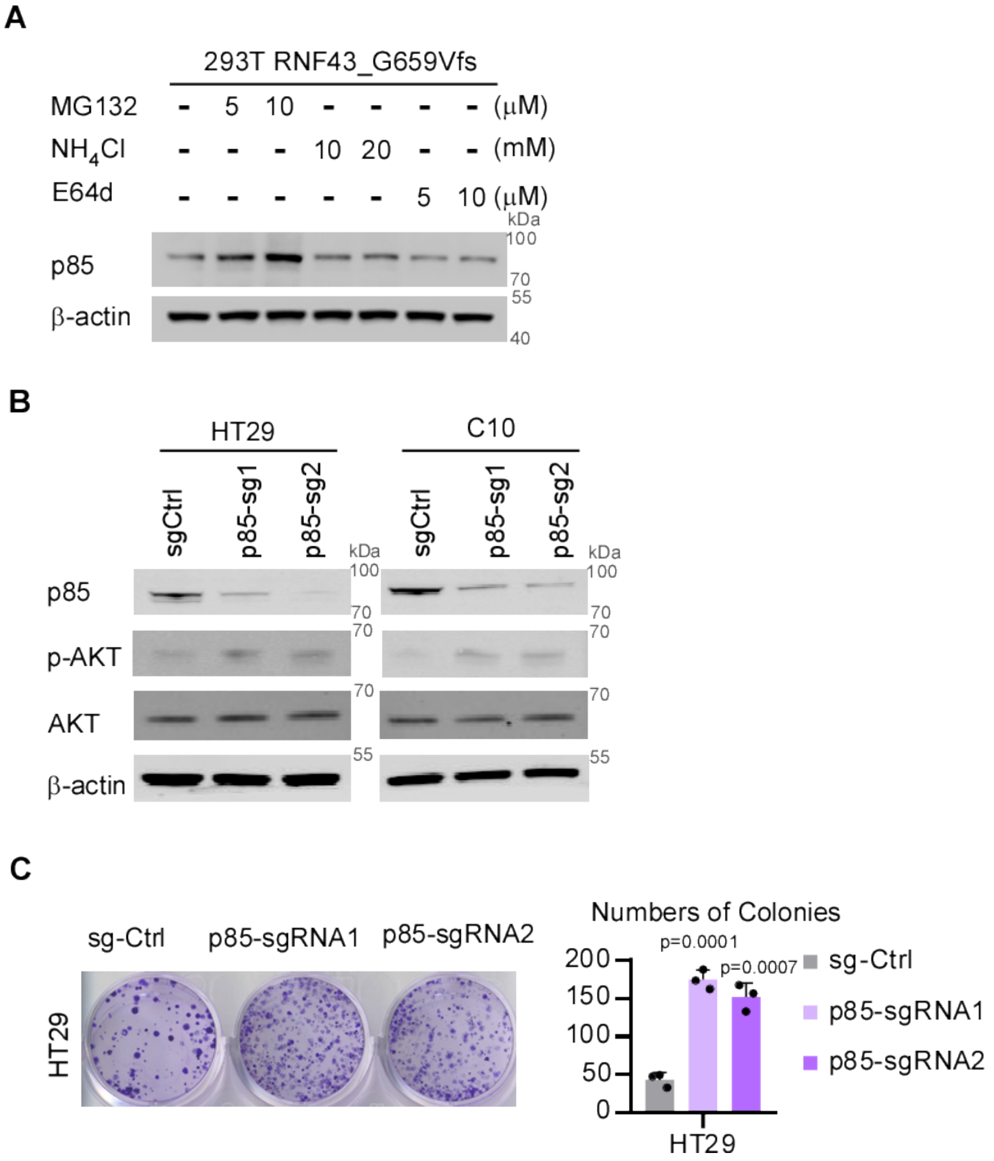

A, Expression of p85 in vector, WT and G659fs 293T cells treated with and without MG132, NH4Cl and E64d at the indicated doses. **B,** p85 knockdown and p-AKT and AKT in HT29 and C10-edited cells. **C,** Colony formation assay in HT29 sg-Ctrl, p85-sgRNA1 (sg1) and p85-sgRNA2 (sg2) with representative image (left) and quantification (right). Data are the mean ± SD. Two-sided Student's t-test (*n* = 3 independent experiments in triplicate). Source data are provided as a Source Data file.

Supplementary Fig. 12 | RNF43_G659Vfs promotes ubiquitination of p85

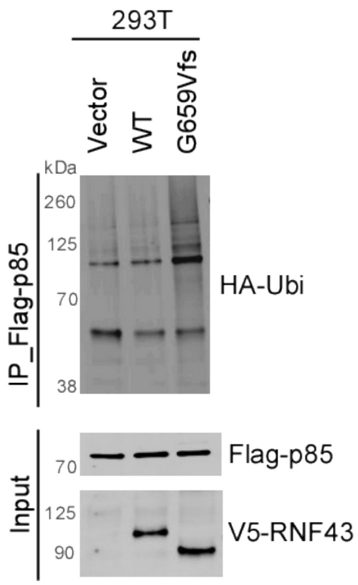

Co-immunoprecipitation assay assessing ubiquitination of Flag-p85 in HEK293T cells expressing V5-tagged vector, *RNF43*-WT, or *RNF43*-G659Vfs*41. Source data are provided as a Source Data file.

Supplementary Fig. 13 | Poly-ubiquitination of p85 in HT29-*RNF43*^{659mut}

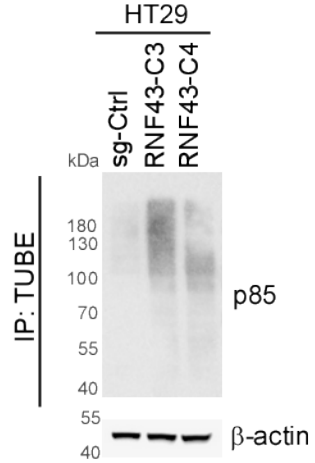

Tandem Ubiquitin Binding Entities (TUBEs) immunoprecipitation from isogenic *RNF43*^{659mut} HT29 cell lysates followed by immunoblotting for p85. Source data are provided as a Source Data file.

Supplementary Fig. 14 | Computational modeling of RNF43 with hypothesized RNF43_659fs/p85 interaction

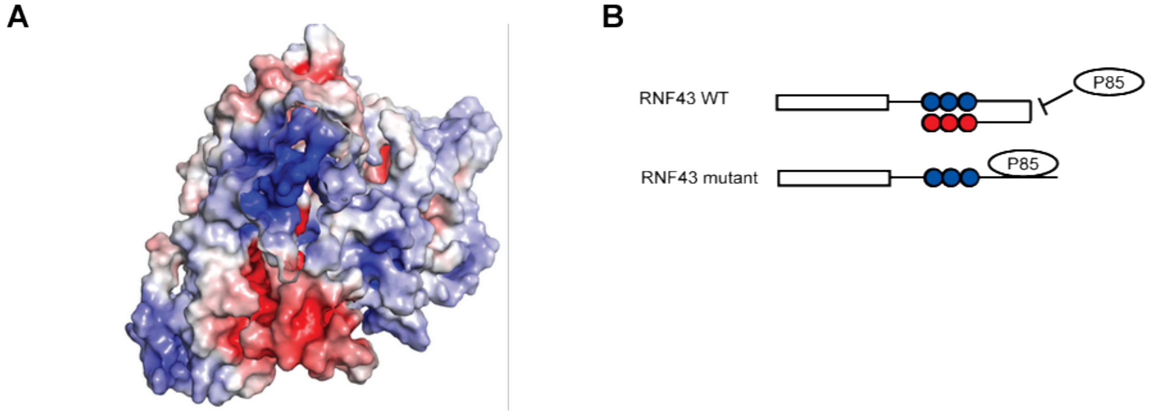

A, Electrostatic view of WT RNF43 model. Electrostatic potential is expressed as a spectrum ranging from -5 kT/e (red) to +5 kT/e (blue). The RNF43 model was predicted by the Zhang server (https://zhanglab.dcmb.med.umich.edu/I-TASSER/). **B,** Schemas of RNF43 wildtype and G659fs interaction with p85. Positively charged region (~aa 551-578) and negatively charged region (~aa 736-781) are shown in blue and red color, respectively.

Supplementary Fig. 15 | Gene Set Enrichment Analysis of HT29 *RNF43*^{659mut}

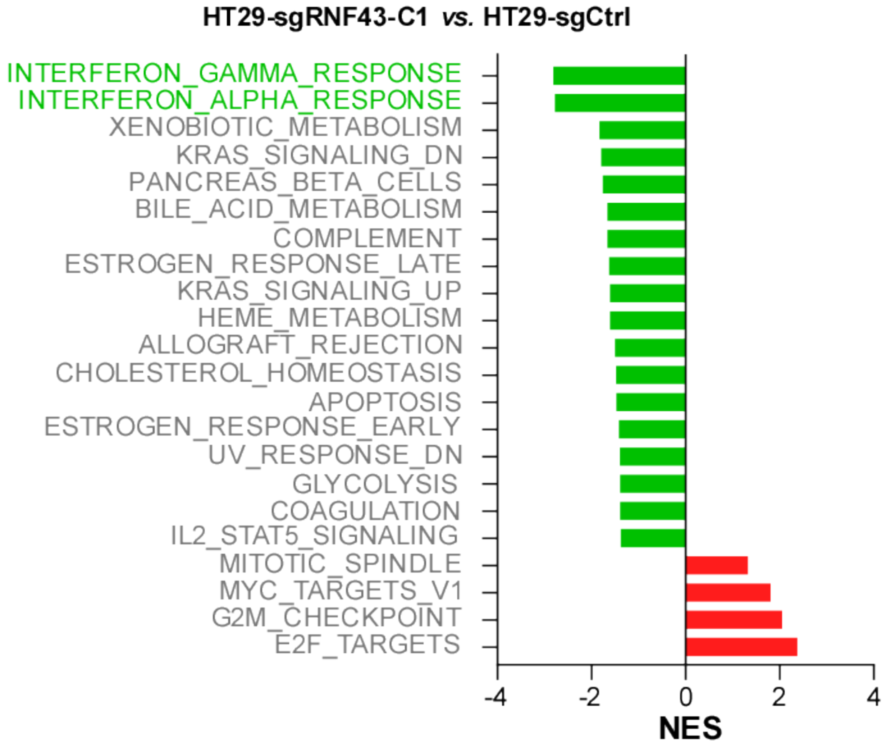

GSEA analysis of HT29 *RNF43*^{659mut} RNA-Seq compared to isogenic control cells. Green bars represent gene sets with decreased expression and red bars represent gene sets with increased expression. Source data are provided as a Source Data file. NES: normalized enrichment score

Supplementary Table 1: mTOR/PI3K drug hits from primary screening (22 selective compounds over *RNF43* WT isogenic cells bolded)		
**Drug Name**	**MOA**	**Phase**
**WYE-125132**	**mTOR inhibitor**	**Preclinical**
**OSI-027**	**mTOR inhibitor**	**Phase 1**
**MLN0128**	**mTOR inhibitor**	**Phase 2**
**torin-1**	**mTOR inhibitor**	**Preclinical**
**torin-2**	**mTOR inhibitor**	**Preclinical**
**AZD2014**	**mTOR inhibitor**	**Phase 2**
**AZD8055**	**mTOR inhibitor**	**Phase 1**
**WYE-354**	**mTOR inhibitor**	**Preclinical**
**XL388**	**mTOR inhibitor**	**Preclinical**
WYE-687	mTOR inhibitor	Preclinical
WAY-600	mTOR inhibitor	Preclinical
**PKI-179**	**mTOR inhibitor, PI3K inhibitor**	**Phase 1**
**NVP-BEZ235**	**mTOR inhibitor, PI3K inhibitor**	**Phase 2**
**SB-2343**	**mTOR inhibitor, PI3K inhibitor**	**Phase 1**
**voxtalisib**	**mTOR inhibitor, PI3K inhibitor**	**Phase 2**
**PF-04691502**	**mTOR inhibitor, PI3K inhibitor**	**Phase 2**
GSK2126458	mTOR inhibitor, PI3K inhibitor	Phase 1
**SAR405**	**PI3K inhibitor**	**Preclinical**
**alpelisib**	**PI3K inhibitor**	**Phase 3**
**CH5132799**	**PI3K inhibitor**	**Phase 1**
**buparlisib**	**PI3K inhibitor**	**Phase 3**
**PIK-93**	**PI3K inhibitor**	**Preclinical**
**copanlisib**	**PI3K inhibitor**	**Launched**
**GDC-0941**	**PI3K inhibitor**	**Phase 2**
**taselisib**	**PI3K inhibitor**	**Phase 3**
ZSTK-474	PI3K inhibitor	Phase 1/Phase 2
CUDC-907	PI3K inhibitor	Phase 2
IPI-145	PI3K inhibitor	Phase 3
PI-828	PI3K inhibitor	Preclinical
LY3023414	PI3K inhibitor, mTOR inhibitor	Phase 2

Supplementary Table 2. Characteristics of Organoid Samples		
Sample	COCA-0235 (Tumor)	5334_N (Normal)
Patient Sex	F	M
Patient Age (yo)	50-60	50-60
Tumor Location	Right Colon	Left colon
MMR status	Deficient	Proficient
Mutations (Tier 1 & 2)	KRAS (p.G13D); MSH2 (p.S473*); RNF43 (p.G659V*41)	N/A

Supplementary Table 3. Primer sequences used for cloning and PCR	
Primer	sequence (5’ to 3’)
RNF43-WT-full length -F	AACACAGGTGTCGTGACGCGGCCACCATGAGTGGTGGCCACCAGCTGCA
RNF43-WT-full length-R	GTTGGTGGCGCCGCTGCATCGAGACCGAGGAGAGGGTTAGGGATCACAGCCTGTTCACAC
RNF43-659fs-full length -F	AACACAGGTGTCGTGACGCGGCCACCATGAGTGGTGGCCACCAGCTGCA
RNF43-659fs-full length -R	GTTGGTGGCGCCGCTGCATCGAGACCGAGGAGAGGGTTAGGGATAGGGGTGTGCCTCTGG
RNF43-659stop terminal -F	AACACAGGTGTCGTGACGCGGCCACCATGAGTGGTGGCCACCAGCTGCA
RNF43-659stop terminal-R	GTTGGTGGCGCCGCTGCATCGAGACCGAGGAGAGGGTTAGGGATCCCCCGCCTTTTCCTC

Supplementary Table 4. Primers used for validation of CRISPR/Cas9 editing by Next Generation Sequencing (NGS) and Sanger Sequencing	
Primer	sequence (5' to 3')
$RNF43^{117mut}$_NGS-F	AAGCCTCCCTAACCCAAGTC
$RNF43^{117mut}$_NGS-R	AGCACAGGGTCTTCTCACAG
$RNF43^{659mut}$_NGS-F	CCACCTTCTCCTGATCAGCA
$RNF43^{659mut}$_NGS-R	AAAATCTGGCAAGCTGGGTG
$RNF43^{659mut}$_Sanger-F	GTCCAGGCCTCCTATTCCTC
$RNF43^{659mut}$_Sanger-R	CACCCACTTCCCTCTGAAAA

Supplementary Table 5. List of antibodies used for immunoblotting and detection				
Antibody	Clone	dilution	Manufacturer catalog number	RRID
β-Actin	8H10D10	1:1000	Cell Signaling Technology #3700	AB_2242334
GAPDH	mAbcam 9484	1:2000	Abcam # ab9484	AB_307274
p-AKT (Ser473)	D9E	1:500	Cell Signaling Technologies #4060T	AB_2315049
AKT	11E7	1:1000	Cell Signaling Technologies #4685S	AB_2225340
4EBP1	236B4	1:1000	Cell Signaling Technologies #2855S	AB_560835
p-4EBP1(Ser65)	174A9	1:1000	Cell Signaling Technology #9456	AB_823413
S6 Ribosomal Protein	5G10	1:1000	Cell Signaling Technology #2217S	AB_331355
p-S6 Ribosomal Protein (Ser235/236)	D57.2.2E	1:1000	Cell Signaling Technology #4858	AB_916156
p85	19H8	1:500	Cell Signaling Technology # 4257S	AB_659889
LRP6	C5C7	1:500	Cell Signaling Technology #2560S	AB_2139329
p-LRP6 (Ser1490)	2568	1:500	Cell Signaling Technology #2568S	AB_2139327
β-Catenin	14/Beta-Catenin	1:3000	BD Biosciences #610153	AB_397554
GSK3β	D5C5Z	1:2000	Cell Signaling Technology #12456S	AB_2636978
p-GSK3β (Ser9)	9336	1:500	Cell Signaling Technology #9336S	AB_331405
CK1	2655	1:2000	Cell Signaling Technology #2655S	AB_2283593
Anti-Ubiquitinylated proteins	FK2	1:1000	Sigma Aldrich #04263	AB_612093
V5-tag	N/A	1:1000	Thermo-Fisher Scientific # R960-25	AB_2556564
IRDye 680CW goat anti-mouse	N/A	1:5000	LI-COR Biosciences #926-68070	AB_10956588
IRDye 800CW goat anti-mouse	N/A	1:5000	LI-COR Biosciences #926-32210	AB_621842
IRDye 800CW goat anti-rabbit	N/A	1:5000	LI-COR Biosciences #926-32211	AB_2224293

Supplementary Table 6. Primers for qRT-PCR	
Primer	sequence (5’ to 3’)
IFIT1 -realtime PCR-F	GCGCTGGGTATGCGATCTC
IFIT1 -realtime PCR-R	CAGCCTGCCTTAGGGGAAG
OAS2 -realtime PCR-F	CTCAGAAGCTGGGTTGGTTTAT
OAS2 -realtime PCR-R	ACCATCTCGTCGATCAGTGTC
RSAD2 -realtime PCR-F	TGGGTGCTTACACCTGCTG
RSAD2 -realtime PCR-R	GAAGTGATAGTTGACGCTGGTT
MX1 -realtime PCR-F	GTTTCCGAAGTGGACATCGCA
MX1 -realtime PCR-R	CTGCACAGGTTGTTCTCAGC
