# Supplementary material for: RNF43 G659fs is an oncogenic colorectal cancer mutation and sensitizes tumor cells to PI3K/mTOR inhibition
Source: Nat Commun. 2022 Jun 8;13:3181. doi: 10.1038/s41467-022-30794-7 (PMC9177965; doi:10.1038/s41467-022-30794-7)
Supplement: Supplementary file 2 — Description of Additional Supplementary Files [file 41467_2022_30794_MOESM2_ESM.pdf]

Description of Additional Supplementary Files

File Name: Supplementary Data 1

Description: Primary drug screening hit list (612 compounds, z-score < -3)

File Name: Supplementary Data 2

Description: Proteomic Analysis of RNF43_G659Vfs for identification of interacting partners ($p < 0.05$)
