# Supplementary material for: RNF43 G659fs is an oncogenic colorectal cancer mutation and sensitizes tumor cells to PI3K/mTOR inhibition
Source: Nat Commun. 2022 Jun 8;13:3181. doi: 10.1038/s41467-022-30794-7 (PMC9177965; doi:10.1038/s41467-022-30794-7)
Supplement: Supplementary file 5 — Reporting Summary [file 41467_2022_30794_MOESM5_ESM.pdf]

nature portfolio

Corresponding author(s): Marios Giannakis

Last updated by author(s): Apr 14, 2022

Reporting Summary

Nature Portfolio wishes to improve the reproducibility of the work that we publish. This form provides structure for consistency and transparency in reporting. For further information on Nature Portfolio policies, see our Editorial Policies and the Editorial Policy Checklist.

Statistics

For all statistical analyses, confirm that the following items are present in the figure legend, table legend, main text, or Methods section.

n/a	Confirmed	
☐	☒	The exact sample size (n) for each experimental group/condition, given as a discrete number and unit of measurement
☐	☒	A statement on whether measurements were taken from distinct samples or whether the same sample was measured repeatedly
☐	☒	The statistical test(s) used AND whether they are one- or two-sided<br>*Only common tests should be described solely by name; describe more complex techniques in the Methods section.*
☒	☐	A description of all covariates tested
☐	☒	A description of any assumptions or corrections, such as tests of normality and adjustment for multiple comparisons
☐	☒	A full description of the statistical parameters including central tendency (e.g. means) or other basic estimates (e.g. regression coefficient) AND variation (e.g. standard deviation) or associated estimates of uncertainty (e.g. confidence intervals)
☐	☒	For null hypothesis testing, the test statistic (e.g. F, t, r) with confidence intervals, effect sizes, degrees of freedom and P value noted<br>*Give P values as exact values whenever suitable.*
☒	☐	For Bayesian analysis, information on the choice of priors and Markov chain Monte Carlo settings
☒	☐	For hierarchical and complex designs, identification of the appropriate level for tests and full reporting of outcomes
☒	☐	Estimates of effect sizes (e.g. Cohen's d, Pearson's r), indicating how they were calculated

Our web collection on statistics for biologists contains articles on many of the points above.

Software and code

Policy information about availability of computer code

Data collection: No software was used for data collection.

Data analysis:
Graphpad Prism (Version 9.1.1)
ImageJ (Version 1.53)
CbioPortal (Version 3.7.15; https://www.cbioportal.org/)
Applied Biosystems QuantStudio (Version 1.3) was used for qRT-PCR analysis.
GSEA (Version 2.0.9) with the hallmark gene sets database (https://www.gsea-msigdb.org/gsea/msigdb/collections.jsp) was used for GSEA experiments.

For manuscripts utilizing custom algorithms or software that are central to the research but not yet described in published literature, software must be made available to editors and reviewers. We strongly encourage code deposition in a community repository (e.g. GitHub). See the Nature Portfolio guidelines for submitting code & software for further information.

Data

Policy information about availability of data

All manuscripts must include a data availability statement. This statement should provide the following information, where applicable:

- Accession codes, unique identifiers, or web links for publicly available datasets
- A description of any restrictions on data availability
- For clinical datasets or third party data, please ensure that the statement adheres to our policy

Data supporting the findings of this study are available within the article the the supplementary information files, and the Source Data file. Source data are provided

with this paper. The Cancer Genome Atlas (TCGA, PanCancer Atlas) and Cancer Cell Line Encyclopedia (Broad, 2019) datasets were accessed used via CBioPortal (https://www.cbioportal.org/). Uniprot human database (https://www.uniprot.org/) was used for identification of proteins by mass spectrometry. GSEA experiments were performed using GSEA 2.0.9 software with the hallmark gene sets database (https://www.gsea-msigdb.org/gsea/msigdb/collections.jsp). The RNA-seq data for this study have been deposited to the GEO public data set under the series GSE179464 (https://www.ncbi.nlm.nih.gov/geo/query/acc.cgi?acc=GSE179464).

Field-specific reporting

Please select the one below that is the best fit for your research. If you are not sure, read the appropriate sections before making your selection.

☒ Life sciences ☐ Behavioural & social sciences ☐ Ecological, evolutionary & environmental sciences

For a reference copy of the document with all sections, see nature.com/documents/nr-reporting-summary-flat.pdf

Life sciences study design

All studies must disclose on these points even when the disclosure is negative.

Sample size	We did not perform sample size calculations and sample sizes were chosen according to accepted standards in the field. Individual data points are shown in each figure or described in the figure legend. Organoid experiments were performed using all available materials. RNA-sequencing was performed with 3 independent replicates as is typical for transcriptomic analysis of cell lines.
Data exclusions	No data was excluded from this study.
Replication	All experiments were repeated three times, unless indicated in the figure legend or methods. Small molecule library screening was performed in duplicate, all other experiments were performed in at least triplicate.
Randomization	All mice were randomly assigned to groups.
Blinding	As most experiments involved comparisons between isogenic cell lines, blinding is unlikely to introduce significant bias and co-authors were not blinded. However, many of the experiments were independently conducted by the authors.

Reporting for specific materials, systems and methods

We require information from authors about some types of materials, experimental systems and methods used in many studies. Here, indicate whether each material, system or method listed is relevant to your study. If you are not sure if a list item applies to your research, read the appropriate section before selecting a response.

Materials & experimental systems

n/a	Involved in the study	
☐	☒	Antibodies
☐	☒	Eukaryotic cell lines
☒	☐	Palaeontology and archaeology
☐	☒	Animals and other organisms
☐	☒	Human research participants
☒	☐	Clinical data
☒	☐	Dual use research of concern

Methods

n/a	Involved in the study	
☒	☐	ChIP-seq
☒	☐	Flow cytometry
☒	☐	MRI-based neuroimaging

Antibodies

Antibodies used

β-Actin (8H10D10), 1:1000, Cell Signaling Technology #3700
GAPDH (mAbcam 9484), 1:2000, Abcam # ab9484
p-AKT (Ser473) (D9E), 1:500, Cell Signaling Technologies #4060T
AKT (11E7), 1:1000, Cell Signaling Technologies #4685S
4EBP1 (236B4), 1:1000, Cell Signaling Technologies #2855S
p-4EBP1(Ser65) (174A9), 1:1000, Cell Signaling Technology #9456
S6 Ribosomal Protein (5G10), 1:1000, Cell Signaling Technology #2217S
p-S6 Ribosomal Protein (Ser235/236) (D57.2.2E), 1:1000, Cell Signaling Technology #4858
p85 (19H8), 1:500, Cell Signaling Technology # 4257S
LRP6 (C5C7), 1:500, Cell Signaling Technology #2560S
p-LRP6 (Ser1490) (2568), 1:500, Cell Signaling Technology #2568S
β-Catenin (14/Beta-Catenin), 1:3000, BD Biosciences #610153
GSK3β (D5C5Z), 1:2000, Cell Signaling Technology #12456S
p-GSK3β (Ser9) (9336), 1:500, Cell Signaling Technology #9336S
CK1 (2655), 1:2000, Cell Signaling Technology #2655S
Anti-Ubiquitinylated proteins (FK2), 1:1000, Sigma Aldrich #04263

	V5-tag, 1:1000, Thermo-Fisher Scientific # R960-25
Validation	All antibodies are validated by the manufacturer as suitable for western blotting. All antibodies were validated in the lab by band specificity and product size in western blot experiments. β-Actin, GAPDH, p-AKT (Ser473), AKT, 4EBP1, S6, p85, LRP6, β-Catenin, GSK3β, CK1 were validated by use of positive control cell lines know to express the target protein based on manufacturer product reference data, previous literature, or CCLE expression data. p85, AKT and V5 antibodies were further validated by showing decreased signal upon RNAi or Crispr knockout of target genes (p85, AKT, RNF43-V5). FK2 antibody was validated by increased signal upon proteosome inhibition across multiple cell lines.

Eukaryotic cell lines

Policy information about cell lines

Cell line source(s)	HT29, HCT116 , LS513 , HEK293T , L-Wnt3a cell lines were obtained from the American Type Culture Collection (ATCC), 293T-HA-RspoI-Fc was obtained from Trevigen (# 3710-001-K) and C10 cell line (ECACC Cat# 12022901) was obtained from the European Collection of Authenticated Cell Cultures (ECACC).
Authentication	Cell lines authenticated by STR profiling.
Mycoplasma contamination	All cell lines tested negative for Mycoplasma contamination.
Commonly misidentified lines (See ICLAC register)	No cell lines used are commonly misidentified.

Animals and other organisms

Policy information about studies involving animals; ARRIVE guidelines recommended for reporting animal research

Laboratory animals	Taconic NCr-Nude (CrTac:NCr-Foxnlnu) female mice at 7 weeks of age were used for cell line xenograft experiments.
Wild animals	No wild animals were used in this study.
Field-collected samples	No field collected samples were used in this study.
Ethics oversight	All animal experiments and study protocols were reviewed and approved by the Dana-Farber Cancer Institute's Animal Care and Usage Committee (IACUC; protocol 20-013), in compliance with the Animal Welfare Act and the Office of Laboratory Welfare (OLAW) of the National Institutes of Health (NIH).

Note that full information on the approval of the study protocol must also be provided in the manuscript.

Human research participants

Policy information about studies involving human research participants

Population characteristics	Tumor organoid:<br>Patient Sex: F<br>Patient Age: 51<br>Tumor Location: Right Colon<br>MMR status: Deficient<br>Tier 1/2 Mutations: KRAS (p.G13D); MSH2 (p.S473*); RNF43 (p.G659V*41)<br><br>Normal Organoid<br>Patient Sex: M<br>Patient Age: 57<br>Tumor Location: Left Colon<br>MMR status: Proficient<br>Tier 1/2 Mutations: None
Recruitment	Participants were enrolled from the Dana-Farber Cancer Institute Gastrointestinal Cancer Center. All new patients to the center were approached for enrollment in the study.
Ethics oversight	The study was approved by the Dana-Farber/Harvard Cancer Center Institutional Review Board. The Dana-Farber Cancer Institute (DFCI) has an approved Federal Wide Assurance (FWA -FWA00001121) on file with the U.S. Department of Health and Human Services (DHHS), Office for Human Research Protections (OHRP) expiring January 21, 2026. The DFCI IRB Organization number is IORG0000035 and expires on September 1, 2023.

Note that full information on the approval of the study protocol must also be provided in the manuscript.
